# Recent Progress on Graphene/Polyaniline Composites for High-performance Supercapacitors

**DOI:** 10.3390/ma12091451

**Published:** 2019-05-05

**Authors:** Xiaodong Hong, Jiawei Fu, Yue Liu, Shanggong Li, Xiaoliang Wang, Wei Dong, Shaobin Yang

**Affiliations:** College of Materials Science and Engineering, Liaoning Technical University, Fuxin 123000, China; fjw1518816615@163.com (J.F.); liuyue471804@163.com (Y.L.); hxdhit@163.com (S.L.); ningke@163.com (X.W.); lgddongwei@163.com (W.D.); yunwen2004@126.com (S.Y.)

**Keywords:** supercapacitors, graphene, polyaniline, electrochemical performance, composite electrodes

## Abstract

Electrode materials are crucial for the electrochemical performance of supercapacitors. In view of the high specific surface area, high conductivity of graphene nanosheets and the high pseudocapacitance of polyaniline (PANI), the combination of graphene with PANI has become a research hotspot. In this work, we summarize the recent advance on the synthesis of PANI and graphene/PANI composites, and their application in supercapacitors. The synthesis of PANI is the basis of preparing graphene/PANI composites, so we first introduce the synthesis methods of PANI. Then, the advances of two dimensional (2D) and three dimensional (3D) graphene/PANI composites are summarized according to the inherent feature of graphene. The 2D composites of pristine graphene and functionalized graphene with PANI are introduced separately; furthermore, the 3D composites are classified into three sections, including flexible graphene/PANI composites, graphene framework based composites, and printable graphene/PANI composites. At last, aiming at solving the current challenges of graphene/PANI composites, we put forward some strategies for preparing high performance graphene/PANI composite electrodes.

## 1. Introduction

With a large consumption of non-renewable fossil energies, the development of new energies is imminent. Among the existing chemical energy sources, supercapacitors have the advantages of fast charging, long life and high cycle stability [1]. However, the performance of supercapacitors depends entirely on the electrode materials. At present, the electrode materials of supercapacitors are mainly divided into two categories. One is carbon based materials with electric double layer feature. These materials usually include graphene, carbon nanotubes, porous carbon, carbon foam, activated carbon, carbon fiber and so on [2]. Their capacitance is mainly determined by the specific surface area of the electrode material, and their specific capacitances are generally ranging from 30 to 200 F g^−1^. Moreover, these materials often show a stable long-term cycling performance. The other electrode materials have pseudocapacitive feature, which achieve the electron transfer and charge storage through redox reaction [3]. Pseudocapacitive materials mainly include conductive polymers, metal oxides, hydroxides, nitrides, sulfides and so on. Among these pseudocapacitive materials, polyaniline (PANI) is a kind of high pseudocapacitance materials with the advantages of low cost, easy synthesis and high conductivity [4]. However, the main shortage of PANI is the poor cycling performance. In order to improve the electrochemical performance of supercapacitors, the combination of carbon based materials with PANI has been become a popular topic, especially in the design and preparation of graphene/PANI composites. In recent years, a lot of works have been reported in the controllable synthesis of PANI and graphene/PANI composites [5]. 

In this review, we mainly summarize the recent advances on the synthesis of PANI and graphene/PANI composites, and their application in supercapacitors. In the first section, we classify the synthesis methods of PANI into five categories, including chemical oxidative method, template method, electrochemical oxidative method, interfacial polymerization and hydrothermal reaction. Then, the research progress of two dimensional (2D) and three dimensional (3D) graphene/PANI composites are introduced respectively. Especially in the third part, we introduce the advance of 3D graphene/PANI composites according to the inherent feature of 3D graphene framework, including freestanding flexible composites, graphene framework based composites, and printable composites. At last, we give a summary and put forward some strategies to prepare high performance graphene/PANI composite electrodes for supercapacitors.

## 2. Synthesis Methods of PANI

Various PANI nanostructures can be synthesized by different methods [6], including chemical oxidative method, template method, electrochemical oxidative method, interfacial polymerization and hydrothermal reaction. Besides chemical methods, a physical method of surface treatment of PANI was also introduced. In this section, we mainly introduce these synthesis methods and the electrochemical performance of various PANI electrodes. 

### 2.1. Chemical Oxidative Method

Chemical oxidation method is widely used for synthesizing PANI and PANI-based composites. Typically, the acidic solution of aniline monomer and ammonium persulfate (APS) solution are mixed rapidly or drop by drop. The polymerization reaction usually occurs in ice-water bath or at room temperature. During a polymerization reaction, the doping process and the oxidation process occur simultaneously. Moreover, the acidic condition is necessary for providing the required pH values and ensuring the doping of PANI backbone skeleton by protonic acid, which endow the electron conductivity of PANI. Through changing the mass ratio of aniline/APS, acid types, reaction temperature/time and surfactants, various PANI nanostructures can be obtained, such as, nanocones, nanowires, nanofibers, nanocorals, nanotubes and nanorods. In this section, we summarize the influence of synthesis conditions on the microstructures and performances of PANI.

**Figure 1:**
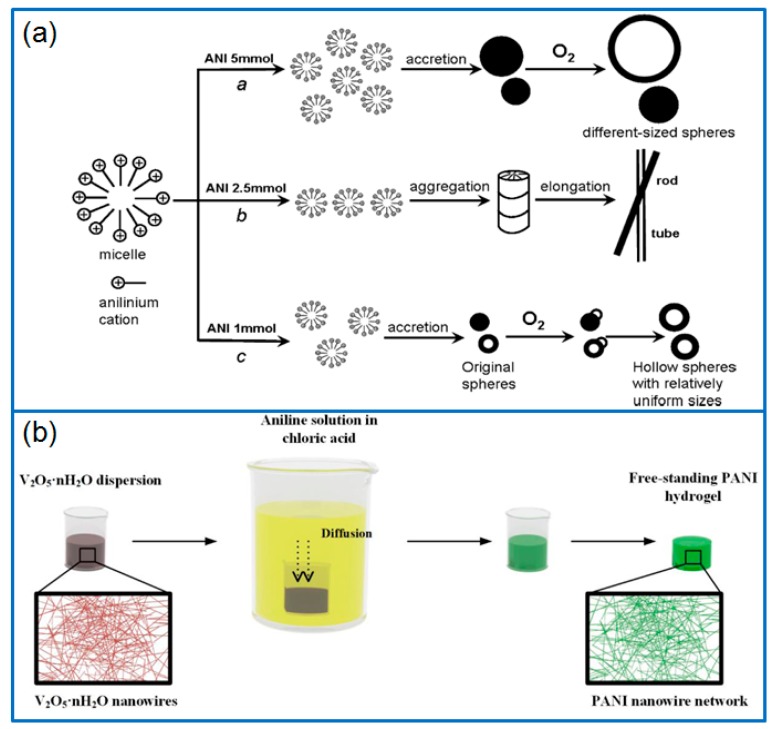
(**a**) Schematic for synthesizing PANI Particles with different morphologies; produced with permission [7]. Copyright 2009, American Chemical Society. (**b**) Schematic illustration for preparing PANI hydrogel using V_2_O_5_ nanowires. Produced with permission [10]. Copyright 2018, American Chemical Society.

*Oxidants*. Except for the popular oxidant of APS, H_2_O_2_ and some metal oxides have also been used for synthesizing PANI. In this field, Yu’s group [7] synthesized well-defined PANI hollow spheres by using H_2_O_2_ as oxidant and Fe^3+^ as catalyst by hydrothermal reaction (140 °C for 6 h). During the reaction process, the formation of PANI hollow spheres was ascribed to the driving force derived from the redox reaction between the benzenoid unit and O_2_, as shown in Figure 1a. Therefore, this work provided a new strategy to prepare PANI hollow spheres without surfactants or sacrificial templates. Du et al. [8] synthesized PANI nanotubes by using MnO_2_ nanotubes as templates and oxidants. To alleviate the volume change of PANI nanotubes during the charge/discharge process, a carbon shell was introduced to encapsulate PANI nanotubes by a hydrothermal method in glucose solution. The results showed that the encapsulated PANI nanotubes had a specific capacitance of 410.5 F g^−1^ at 1 A g^−1^, and the cycle stability was much better than that of PANI nanotubes. In addition, Tabrizi et al. [9] synthesized PANI nanocones on graphene oxide (GO) sheets by using V_2_O_5_ oxidant, followed by using APS oxidant to grow PANI nanoarrays on GO sheets (PANI/GO-V-APS). In order to compare the electrochemical performance, GO/PANI nanocomposites were prepared by using APS and V_2_O_5_ separately. The results showed that the PANI/GO-V-APS electrode had a higher specific capacitance of 712 F g^−1^ at 0.5 A g^−1^, and a higher capacitance retention rate of 83% after 6000 cycles. In addition, V_2_O_5_ oxidant can also be used alone to synthesize PANI hydrogels. Bai group [10] selected vanadium pentoxide hydrate (V_2_O_5_·nH_2_O) nanowires as both sacrifice template and the oxidant, and synthesized pure PANI hydrogels consisted of PANI ultrathin nanofibers (Figure 1b). During a reaction, the aniline was oxidized rapidly by V_2_O_5_·nH_2_O nanowires to form ultrathin PANI nanofibers. Meanwhile, V_2_O_5_·nH_2_O was spontaneously removed by forming soluble salts, and the 3D assembly process led to the quick gelation of PANI nanowires. As a supercapacitor electrode, the PANI hydrogels delivered a specific capacitance of 636 F g^−1^, and a high capacitance retention of ~83% after 10,000 cycles. Therefore, compared to traditional APS oxidant, some metal oxides show a dual function of oxidant and template in the synthesis of PANI, and also enhance the electrochemical performance of PANI.

*Types of acids*. During polymerization of PANI, the acidic condition ensures the doping of PANI backbone skeleton, and also endows the electron conductivity of PANI. Therefore, the acid types have an important influence on the microstructure and electrochemical performance of PANI nanostructures [11,12,13]. Tabrizi et al. [14] investigated the effect of the acid types including H_2_SO_4_, HCl, HClO_4_, and *p*-toluenesulfonic acid (PTSA) on the performance of PANI/GO composite. The PANI/GO composite with well-defined PANI nanoarrays synthesized in H_2_SO_4_ had the highest specific surface area and porosity, which delivered the highest specific capacitance (727 F g^−1^) among these composites.

*Reaction temperature*. The polymerization temperature of PANI was often controlled at room temperature or 0 °C in ice-water bath. However, in order to explore the effect of reaction temperature, Yuan et al. [15] reported a two-step method to synthesize PANI under two successive low-temperature of −20 °C and room temperature of 30 °C. With this special method, a bimodal PANI was synthesized with a coral-like structure containing thorns and interconnected nanowires. This optimized PANI delivered a superior electrochemical performance than that of the composite prepared using the traditional method, with a high specific capacitance of 689 F g^−1^ at 1 A g^−1^.

### 2.2. Template Method

During a synthesis process of PANI, various hard templates and soft templates (surfactants) are usually adopted to control the morphology and dimension of PANI. 

**Figure 2:**
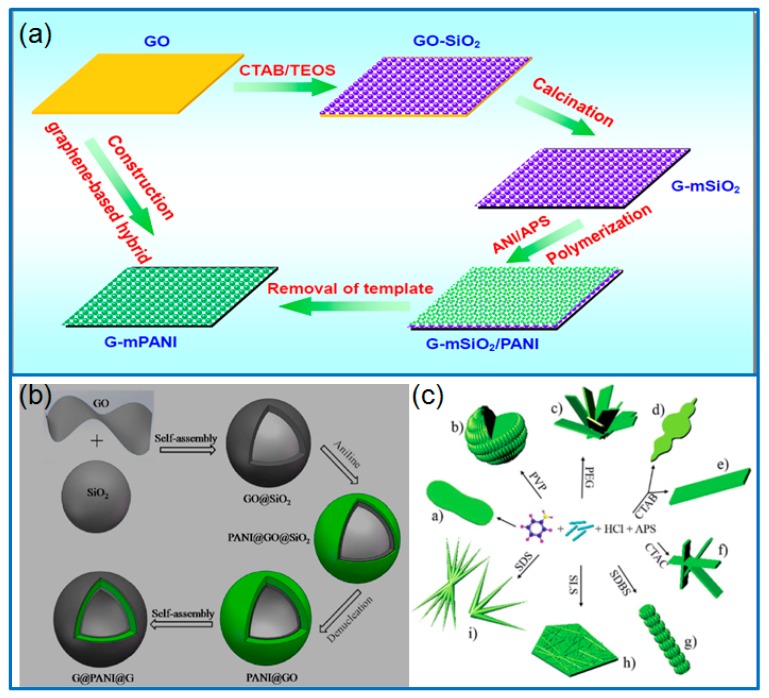
(**a**) Schematic for preparing G-mPANI hybrid material by using SiO_2_ template; produced with permission [21]. Copyright 2015, American Chemical Society. (**b**) Preparation of sandwiched G@PANI@G hollow sphere; produced with permission [22]. Copyright 2013, Elsevier. (**c**) Synthesis of different PANI nanostructures using different surfactants. Produced with permission [24]. Copyright 2017, Royal Society of Chemistry.

*Hard templates*. Polystyrene (PS) microspheres, polymethyl methacrylate (PMMA) particles and silica spheres are commonly served as hard templates. By using PS spheres templates, urchin-like hollow PANI microspheres were synthesized [16,17]. In addition, Wang et al. [18] synthesized urchin-like PANI microspheres based on the template of sulfonated polystyrene microspheres. As supercapacitor electrode, the urchin-like PANI microspheres exhibited a specific capacitance of 435 F g^−1^ at 10 mV s^−1^. Based on the template of negatively charged sulfonated PS spheres, Luo et al. [19] prepared reduced graphene oxide (rGO)-PANI hollow sphere (HS) by a layer-by-layer (LBL) assembly method, in which, the positively charged PANI firstly wrapped PS spheres, then adsorbed negatively charged rGO, and the cycles of alternative assembly would decide the layer thickness of hollow spheres. The rGO-PANI hollow spheres were obtained after dissolving PS templates in THF. As supercapacitor electrodes, the specific capacitance of rGO-PANI hollow spheres reached 381 F g^−1^ at 4 A g^−1^, much higher than that of stacked LBL film of rGO-PANI (251 F g^−1^). Similar to PS spheres, PMMA colloidal particles were used as templates to achieve the self-assembly of GO nanosheets on the particles surface. After growing PANI and removing the PMMA templates, 3D hollow balls consisted of graphene and PANI hybrid (3D-HBGP) were obtained successfully [20]. The 3D-HBGP electrode had a high specific capacitance of 331 F g^−1^ at 1 A g^−1^ in a three-electrode system. Moreover, when assembled in a flexible supercapacitor, the 3D-HBGP composite delivered a capacitance of 182 F g^−1^ at the bending state. Besides polymer microspheres, positively charged SiO_2_ spheres are also served as hard templates for electrostatic adsorbing negatively charged GO, and then generate PANI on the surface of GO@SiO_2_. The hollow PANI@GO spheres were obtained after etching the SiO_2_. Finally, G@PANI@G hollow spheres were prepared after repeatedly wrapping and a reduction process (Figure 2b) [21]. The hollow sandwiched composite electrode exhibited a high capacitance of 682.75 F g^−1^ at 0.5 A g^−1^ and a good cycling stability (87.6% after 10,000 cycles). In another work, Fan et al. [22] synthesized graphene-mesoporous silica (G-mSiO_2_) as templates, then in-situ polymerized PANI on the templates. After removing m-SiO_2_ by rinsing in hydrofluoric acid, the mesoporous G-mPANI composite was obtained with graphene conductive network (Figure 2a). The composite electrode had a specific capacitance of 749 F g^−1^ (0.5 A g^−1^). Beside spherical templates, Tabar et al. [23] prepared PANI hollow fibers (PANI-HF) using electrospun polyacrylonitrile (PAN) nanofibers template. In order to further improve the electrochemical performance of PANI, the PANI-HF was decorated with rGO sheets. The prepared PANI-HF/rGO hybrid composite delivered a specific capacitance of 449 F g^−1^, much higher than that of PANI-HF (425 F g^−1^). Moreover, the capacitance retention rate of PANI-HF/rGO hybrid was 84% after cycling for 2000 cycles. Based on the above findings, various hard templates are essential for improving the electrochemical performance of PANI and its composites by increasing the dispersion and specific surface area of PANI. 

*Soft templates*. In the synthesis of PANI, surfactants are also named as soft templates. Using different surfactants, Ma et al. [24] synthesized various PANI nanostructures including roses, spheres, cloud-like and rhombic plates, columns, layered flowers, blocks and dendrites in a low concentration HCl solution (Figure 2c). The multidimensional (MD) nanostructures of PANI were formed under the synergistic interaction of self-assembly processes and soft templates, and the PANI nanostructures had an important effect on the electrochemical performance of supercapacitor. Among these MD nanostructures, the layered flowers-like PANI showed the highest specific capacitance of 272 F g^−1^ at 1.0 A g^−1^. 

Besides short-chain surfactants, some flexible polymers including polyvinyl alcohol (PVA) and polyethylene glycol (PEG) were adopted to prepare functionalized PANI. In order to achieve the combination of soft hydrophilic polymer and rigid conductive polymer [25], Li et al. [26] synthesized PANI supramolecular hydrogels through a supramolecular strategy of cross-linking. In which, PVA serving as soft polymer and PANI as the rigid polymer, and PANI-PVA hydrogel (PPH) was prepared by crosslinking PVA and PANI with boronic acid groups. The PPH showed a superior electrochemical performance and mechanical durability in a flexible solid-state supercapacitor. Among long-chain modifiers, a long-chain polyethylene glycol segment containing protonic acid (NPES) was used to adjust the flowability of PANI at a room temperature [27]. Through changing the doping ratio of NPES to PANI, the flowability of self-suspended PANI could be adjusted easily. Moreover, the self-suspended PANI can be dissolved in water, chloroform, acetone, and N-methylpyrrolidinone (NMP). Therefore, soft templates are mainly used for adjusting the microstructures, and the feature of flexibility or hardness of PANI. 

### 2.3. Electrochemical Oxidative Method

In an electrochemical oxidative process, a three-electrode cell is required and equipped with a reference electrode, a counter electrode and a working electrode in an acidic solution of aniline. Constant potential or current method is commonly used to achieve the electrochemical polymerization of aniline on the working electrode. With this method, the PANI powder or flexible film can be obtained on the surface of electrode. 

In this field, Li et al. [28] adopted Au/polycarbonate (PC) membrane as a template and electrodeposited orderly PANI nanotubes array film through a cyclic-voltammetry method. Due to the flexible feature of the Au/PC template, as-prepared PANI film could be directly used for flexible all-solid-state supercapacitors. By changing the deposition cycle numbers, the maximum areal capacitance reached 237.5 mF cm^−2^ at 10 mV s^−1^. In another work, a gold electrode was used to electrodeposit PANI film in different dopants [29], and the electrochemical performance of PANI (H_2_SO_4_)-EB film was also tested in corresponding dopant anions. The PANI film deposited in oxalic acid exhibited the best electrochemical performance among the candidate acids including H_2_SO_4_, HClO_4_, H_3_PO_4_, HNO_3_, *p*-toluene sulfonic acid (PTSA) and dodecyl benzene sulfonic acid (DBSA). 

Besides metal or commercial working electrodes, some freestanding porous conductive carbon-based materials are designed for depositing PANI. In the design of working electrode, Zhang et al. [30] first prepared porous tubular carbon (PTC) with mesopores and micropores structure, and coated it on nickel foam as a working electrode, then electrodeposited PANI nanowires at a constant potential. For the synergistic effect of porous PTC and PANI nanowires, the PTC/PANI composite electrode showed a specific capacitance of 477.7 F g^−1^ (1 A g^−1^) and capacitance retention of 91% for 5000 cycles. 

Besides the one-step deposition method for growing PANI, a two-step electrochemical deposition method was also reported. For example, Shabani-Nooshabdi et al. [31] prepared graphene-PANI nanocomposites with a two-step electrodeposition method. In the first step, rGO film was formed on the surface of glassy carbon electrode (GCE) electrode by electrochemical reduction of GO, then the PANI was electrodeposited on its surface by cyclic voltammetry method. The composite electrode combined the synergistic effect of conductive rGO and highly pseudocapacitive PANI, which delivered a maximum specific capacitance of 1084 F g^−1^ at 3.22 mA cm^−2^ in 1M H_2_SO_4_. In another work, Yu et al. [32] deposited rGO and PANI separately on a flexible stainless steel fabric (SSF) substrate to prepare a sandwiched SSF/rGO/PANI hybrid electrode. When assembled in a flexible all-solid-state supercapacitor, the hybrid electrode delivered a high specific capacitance of 1506.6 mF cm^−2^, and a stable cycling stability (92% for 5000 cycles).

### 2.4. Interfacial Polymerization 

Aniline monomer was dissolved in organic solvent, such as carbon tetrachloride, chloroform, benzene and toluene, etc. Meanwhile, the oxidant was dissolved in water or acid solution. After two solutions are slowly poured into a reactor, an organic/water interface was gradually formed for the immiscible behavior, and the whole polymerization reaction took place at the interface of organic solvent/water. PANI is hydrophilic, so the produced PANI diffused into the aqueous phase, and the product concentration at the interface decreased gradually, which ensured the continuous polymerization of aniline monomers. After the reaction completed, the dark green PANI could be obtained through filtration, rinsing and drying. With this method, Kaner group [33] synthesized PANI nanofibers in different acids without any functional dopants or templates. The average diameter of PANI nanofibers ranged from 30 nm to 120 nm. Souza et al. [34] developed a one-pot reaction to prepare graphene/PANI hybrid film, in which, the aniline and graphene were dispersed in benzene, and the oxidant was dissolved in water. After the aniline monomer polymerized, the polymerized PANI film would self-assemble with graphene in the water/benzene interface to produce graphene/PANI composite film. This film could be transferred to various substrates. When served as an electrode in a three-electrode system, the graphene/PANI composite film exhibited a specific capacitance of 267.2 F cm^−3^. Moreover, the film could be used to prepare flexible all-solid supercapacitor device. Compared to chemical oxidative and electrochemical oxidative method, interfacial polymerization involves a self-assembly process, and the usage of organic solvents would cause some drawbacks such as environmental problem and complicated treatments.

### 2.5. Hydrothermal Method

Hydrothermal reaction is a simple and efficient method to prepare cross-linked PANI and graphene/PANI composite. Feng et al. [35] synthesized graphene/PANI nanocomposites by hydrothermally treating the suspension of GO, aniline and APS at 140 °C for 12 h. Before hydrothermal treatment, the ultrasonic treatment of the mixed suspension effectively induced the morphology change of PANI from nanowires to nanocones. Compared with graphene/PANI nanocones composite, the electrode of graphene/PANI nanowires showed a higher specific capacitance (724.6 F g^−1^). In another work, using camphor sulfonic acid as the dopant and dioctyl sulfosuccinate sodium salt as soft template, Guo et al. [36] prepared flexible 3D cross-linked PANI through a hydrothermal reaction at 130 °C for 6 h. The fishnet-shaped PANI electrode exhibited a specific capacitance of 601 F g^−1^ at 0.5 A g^−1^. 

### 2.6. Surface Treatment of PANI

Besides chemical methods for synthesizing PANI, a physical method has also been reported to improve the electrochemical performance of PANI by increasing its specific surface area. For example, Xu et al. [37] soaked the PANI nanofibers in chloroform (CHCl_3_) and stirred for 24 h to prepare a CHCl_3_-treated PANI (HSSA-PANI). The specific surface area and pore volume of treated PANI increased to 817.3 m^2^ g^−1^ and 0.6 cm^3^ g^−1^ from its original values of 33.6 m^2^ g^−1^ and 0.2 cm^3^ g^−1^. Due to the higher specific surface area and pore structure, the HSSA-PANI electrode exhibited an excellent rate capability, with a capacity retention rate of 90% when the current density increased from 5.0 to 30 A g^−1^.

## 3. Two Dimensional Graphene/PANI Composites

Compared to PANI, graphene/PANI composites overcome the poor cycling performance of PANI electrodes, and achieve the synergistic effect of electric double layer and pseudocapacitive feature. According to the feature of graphene, we classify 2D graphene/PANI composites into pristine graphene/PANI composites and functionalized graphene/PANI composites.

### 3.1. Pristine Graphene/PANI Composites 

When synthesizing PANI, GO or rGO sheets are dispersed in the acidic solution of aniline, and the oxidant solution is dropped to induce the polymerization reaction of aniline. In order to further improve the conductivity of composite, the GO sheets are often reduced after growing PANI. Followed this method, Zhang et al. [38] prepared uniform PANI fibers coated graphene nanocomposite and discussed the influence of the ratios of graphene/PANI on their electrochemical performance. The PAG80 composite with aniline/GO mass ratio of 80% had the highest specific capacitance of 480 F g^−1^ at 0.1 A g^−1^. 

**Figure 3:**
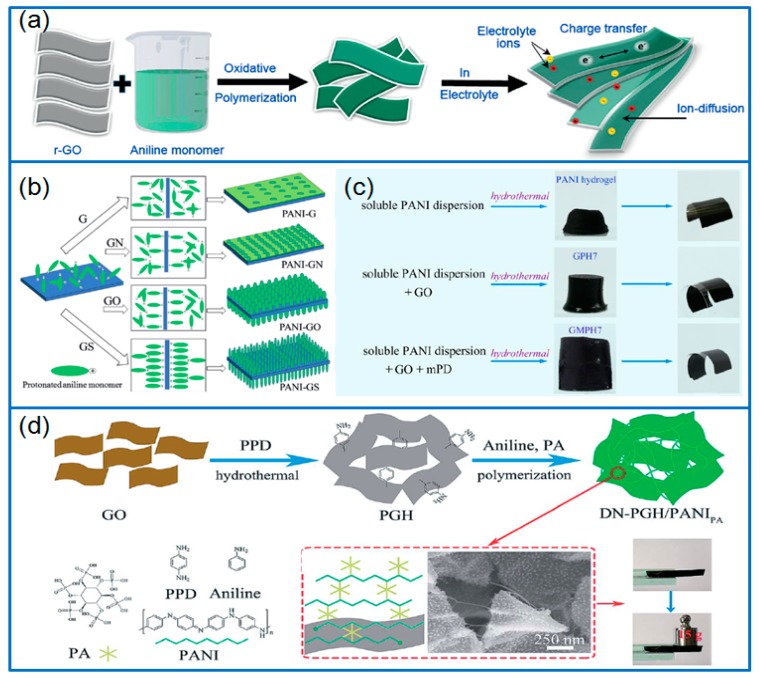
(**a**) Schematic for growing PANI on rGO flakes; produced with permission [39]. Copyright 2014, Wiley-VCH. (**b**) Growing PANI on the graphene surfaces with different functional groups; produced with permission [46]. Copyright 2015, Wiley-VCH. (**c**) preparation of PANI and PANI/graphene hydrogel by adding modifier; produced with permission [47]. Copyright 2018, Royal Society of Chemistry. (**d**) Preparation of stiff DN-PGH/PANI_PA_ hydrogel; produced with permission [48]. Copyright 2018, Royal Society of Chemistry.

In addition, GO sheets can be reduced firstly by using hydrazine hydrate, and then generated PANI [39]. With this method, PANI nanoparticles were uniformly coated on the rGO surface and effectively overcome the stacking of rGO sheets (Figure 3a). As-prepared rGO-PANI electrode exhibited a capacitance of 286 F g^−1^ and capacitance retention of 94% for 2000 cycles. In the preparation of graphene/PANI composites, which are different from a single GO or rGO substrates, Zhang et al. [40] prepared a homogenous suspension of GO/pristine graphene (PG) nanosheets by mixing GO and PG, then growing PANI nanoarrays on GO/PG nanosheets with chemical oxidative polymerization. The GO/PG/PANI ternary composite had a specific capacitance of 793.7 F g^−1^ (1 A g^−1^) in a three-electrode system and 564.2 F g^−1^ (2 A g^−1^) in a sandwich symmetric system, these capacitances were much higher than those of binary composites. 

Compared to traditional graphene, graphene quantum dots (GQDs) have a larger surface area, conductivity and good dispersion in solvents, which can be prepared by cutting GO flakes in H_2_O_2_ [41]. Based on the GODs template, nanofibrous or nanotubular GQDs-PANI composites were prepared with a chemical oxidation method [42]. This novel GODs-based composite delivered a high specific capacitance of 1044 F g^−1^ at 1 A g^−1^, with capacitance retention of 80.1% for 3000 cycles. In addition, porous graphene microspheres (GMS) have a better conductivity than graphene sheets, which can be prepared by spray drying of GO suspension. Serving as conductive frameworks and porous substrates, the GMS was employed to grow PANI nanowire arrays by in-situ polymerization [43]. The microspherical PANI/GMS composite had a good dispersibility and porous structure, which facilitated the diffusion of the ions. The microspherical composite electrode exhibited a specific capacitance of 338 F g^−1^ at 20 mV s^−1^ and capacitance retention of 87.4% for 10,000 cycles. In another similar work, sheet-like PANI/GO composites were firstly synthesized by polymerizing aniline on GO sheets, then microspherical PANI/G composites (PANI/G-MS) were prepared by spray-drying and chemical reduction [44]. Due to the numerous channels in spherical particles and uniform PANI particles on the graphene surface, the PANI/G-MS composite showed a high capacitance of 596.2 and 447.5 F g^−1^ at 0.5 and 20 A g^−1^, respectively.

Besides chemical oxidative method, physical mixing is a simple method for preparing graphene/PANI composites, which includes solution mixing and physical mixing. Some flexible graphene/PANI films can be prepared by filtering the mixed solution of PANI and GO/rGO suspension, and corresponding works will be introduced in the section of freestanding flexible composites. In the field of physical mixing, Chang et al. [45] synthesized particle-deposited tube-like PANI by chemical oxidative polymerization, and prepared the PANI/GO composite by mixing the powders of PANI and GO in ethanol. Through adjusting the ratio of PANI and GO, the optimized PANI/GO composite electrode exhibited a specific capacitance of 475 F g^−1^, and capacitance retention of 90% after 2000 cycles.

### 3.2. Functionalized Graphene/PANI Composites

GO contains abundant oxygen-containing functional groups, such as, carboxyl, hydroxyl and epoxy, which ensure a good hydrophilicity of GO. In order to synthesize high performance graphene/PANI nanocomposites, GO sheets are often decorated with various functional groups by chemical modification [49] or noncovalent functionalization [50]. In this section, we mainly introduce the preparation and performance of functionalized graphene/PANI composites. 

In order to disclose the effect of functional groups of graphene surface on the electrochemical performance of graphene/PANI composites, Liu et al. [46] selected four types of graphene as carriers, including GO, rGO, sulfonated graphene (GS) and aminated graphene (GN), then prepared various structured nanocomposites (Figure 3b). Among these composites, PANI-GS composite with PANI nanorods had a larger specific surface area, and the sulfonic acid groups also facilitated the redox reaction of the PANI. Therefore, PANI-GS composite delivered the largest specific capacitance of 863.2 F g^−1^ at 0.2 A g^−1^. 

Chemically grafted GO sheets have been widely reported and exhibited much more active sites than pristine graphene. In this field, Liu et al. [51] synthesized PANI nanorods arrays on the graphene sheets by using the amino-functionalized graphene sheets (AFG) as initiators. Due to the covalent bonding interaction between PANI chains and graphene, and the hierarchical nanostructure of PANI nanorods, the composite delivered a high specific capacitance of 1295 F g^−1^ at 1 A g^−1^. Li et al. [52] prepared a covalently-grafted PANI/GO nanocomposite by initiating the polymerization of aniline on the aniline-functionalized GO. The PANI/GO facilitated the ion diffusion and redox reaction of PANI, and showed a much higher specific capacitance (442 F g^−1^) and better cycling stability than that of pristine PANI. In another work [53], *p*-aniline groups were grafted on the graphene (G) substrate by *p*-phenylenediamine diazotisation reaction, and then PANI nanofibers were polymerized on the functionalized graphene sheets. As-prepared a-G-PANI200 composite (G/PANI of 1:200) had a capacitance of 422 F g^−1^ at 1 A g^−1^. 

As a chemical mediator, *p*-phenylenediamine (PPD) was used to adjust the PANI microstructures from dendritic to long fibrous, and also served as a nitrogen source to prepare nitrogen-doped graphene. In Luo’s work [54], long PANI nanofiber/N-doped graphene hydrogels (PNGH) was prepared in the presence of PPD mediators by chemical oxidative method and hydrothermal treatment. Through a comparison with various samples, the PNGH hydrogels had the largest compressive strength (75.8 KPa) and the highest specific capacitances of 610 F g^−1^ at 1 A g^−1^. Based on PPD mediator, Bulin et al. [55] prepared PPD functionalized graphene aerogel (GA) by diazonium reaction, followed by grafting PANI to prepare freestanding functionalized GA/PANI composite. The mediators effectively improved the interface bonding between PANI and GA. As a freestanding working electrode in a three-electrode system, the functionalized GA/PANI composite showed a higher specific capacitance (810 F g^−1^), and more stable cycling performance (83.2% of retention after 10,000 cycles) than that of unmodified GA/PANI composite.

Similar to PPD, *m*-phenylenediamine (mPD) was also served as a mediator in preparing N-doped graphene/PANI hydrogels (GMPH7, Figure 3c) [47]. The mPD effectively alleviated the chemical interaction between PANI and GO, and maintained the inherent conjugated structure of PANI [56,57,58]. Therefore, the GMPH7 electrode showed an improved electrochemical performance, with a specific capacitance of 514.3 F g^−1^. Moreover, when assembled in a flexible solid-state supercapacitor, the GMPH7 electrodes delivered a large areal specific capacitance of 584.7 mF cm^−2^. 

Except for a single mediator, two mediators can be used for achieving different functions [48]. For example, PPD was used to prepare functionalized graphene hydrogel (PGH), and then PANI was polymerized on PGH under the presence of phytic acid (PA) to prepare stiff graphene/PANI hydrogels (Figure 3d). Instead of flexible graphene hydrogels, the PGH/PANI hydrogels had a double-crosslinked network and showed a strong mechanical strength, with a tensile strength of 1.39 MPa and ruptured elongation of 0.42%. When assembled in a symmetric supercapacitor, the stiff hydrogels electrodes delivered a specific capacitance of 3488.3 mF cm^−2^ and 872 F cm^−3^. In addition, amino-triazine (AT) functionalized rGO (ATrGO) was prepared by adding 2, 4, 6-Trichloro-[1, 3, 5] triazine (TCTA) and PDA [59]. The special “V-type” amino functionalized rGO achieved the supramolecular assembly between PANI chains. Based on this “V-type” functionalized rGO, a flower-like PANI-ATrGO composite was prepared and exhibited a remarkable specific capacitance of 1510 F g^−1^ at 1 A g^−1^, much higher than that of pristine PANI electrode (487 F g^−1^).

Besides chemical grafting methods, hydrothermal reaction can be used to functionalize rGO. Jin et al. [60] prepared PANI/GO composite films by filtering the mixture of GO and the GO polymerized with PANI nanofibers. After hydrothermal treated in thiourea solution for 10 h at 180 °C, the sulfur functionalized PANI/FrGO composite films were prepared. The flexible film electrode exhibited a specific capacitance of 692.0 F g^−1^, and a capacitance of 324.4 F g^−1^ in all-solid-state supercapacitor at 1 A g^−1^.

Besides decorating graphene by chemical grafting, sulfonated triazine (ST) was used to decorate graphene by hydrogen bonding and *p*-*p* stacking interactions [61]. Moreover, ST functionalized graphene sheets (STGNS) had a good dispersion in water. The 3D PANI-STGNS hybrid materials were obtained by filtering the suspension of PANI nanorods coated STGNS. Through optimizing the loading of STGNS, the PANI-STGNS10 (with 10% STGNS) composite exhibited a high specific capacitance of 1225 F g^−1^ at 1 A g^−1^. Considering the hydrogen bonds formed between tannic acid (TA) and aniline, TA was used as mediator to control the formation of PANI nanoparticles [62]. In the presence of TA, the aniline monomers were polymerized on the GO sheets. The flexible rGO/PANI nanocomposite film was prepared by reducing the GO/PANI suspension. As electrodes for flexible all-solid-state supercapacitors, the nanocomposite film exhibited a specific capacitance of 0.92 F cm^−2^ or 1314.3 F cm^−3^. Similar to tannic acid (TA), pyrenebutyric acid (PBA) was also served as mediators. Kumari et al. [63] prepared PBA functionalized rGO, and then grew PANI by interfacial polymerization. After removing the PBA surface modifier, the porous PANI-rGO composite was obtained and delivered a specific capacitance of 630 F g^−1^ (0.5 A g^−1^) in a three-electrode supercapacitor.

Therefore, compared to directly growing PANI or mixing PANI with pristine graphene, the decorated graphene usually form chemical bonds with polymerized PANI, and the resulting functionalized graphene/PANI composites exhibit an enhanced electrochemical performance. 

## 4. Three Dimensional Graphene/PANI Composites

Compared to 2D graphene/PANI composites, 3D graphene/PANI composites have received much more attention for their freestanding feature, high conductivity, abundant porous structure and high specific surface area. Some graphene framework or hydrogels have been widely employed as substrates for growing PANI nanostructures. According to the inherent feature and preparation method of graphene frameworks, 3D graphene/PANI composites are classified into three parts, freestanding flexible composites, graphene framework based composites, and printable composites.

### 4.1. Freestanding Flexible Composites 

With an increasing requirement for portable and wearable energy storage devices, the lightweight flexible supercapacitors are widely reported in recent years. Some conductive carbonaceous materials including carbon cloths, carbon fibers, carbon nanotubes and graphene, are adopted to hybridize with pseudocapacitive materials to fabricate high performance flexible composite electrodes. In this section, some design strategies of flexible graphene/PANI composites and their electrochemical performance are summarized. 

A flexible composite film can be simply prepared by vacuum filtration of the mixture suspension. By this method, Wu et al. [64] prepared flexible paper-like film composed of PANI nanofibers (PANI-NFs) and chemically converted graphene (CCG). The PANI-NFs sandwiched between graphene layers effectively prevented the stacking of graphene nanosheets. When assembled in a two-electrode supercapacitor, the flexible composite film electrodes delivered a capacitance of 210 F g^−1^ at 0.3 A g^−1^. In addition, Du et al. [65] prepared freestanding oriented graphene hydrogel (OGH) films by vacuum filtration, and then polymerized PANI on the OGH to obtain OGH-PANI composite film for flexible solid-state supercapacitors. This film electrode showed a specific capacitance of 530 F g^−1^ and capacitance retention of 80% after 10,000 cycles. Furthermore, the flexible electrode retained ~100% capacitance retention after 180° bending for 250 cycles.

**Figure 4:**
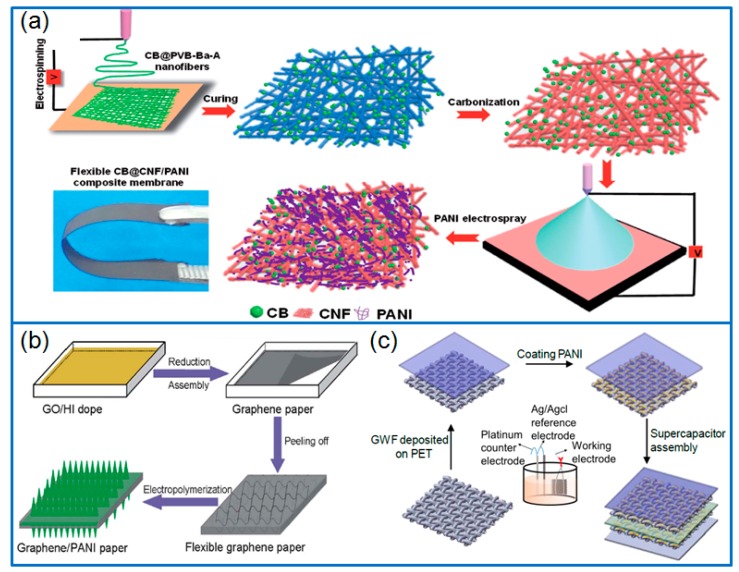
(**a**) Schematic for fabricating flexible CB@CNF/PANI composite film; produced with permission [67]. Copyright 2017, Wiley-VCH. (**b**) The preparation process of graphene-PANI paper; produced with permission [68]. Copyright 2019, Royal Society of Chemistry. (**c**) Preparation of GWF + PANI supercapacitor; produced with permission [69]. Copyright 2019, Royal Society of Chemistry.

When preparing flexible graphene based composites, some modifiers are also used for improving the pore structure, conductivity and dispersion. For example, cellulose nanofibers (CNF) were added into graphite nanoplatelets (GNP) suspension and prepared freestanding flexible substrates with thickness of 10 μm. The CNF/GNP substrate was immersed in aniline solution and generated with worm-like PANI nanorods through in situ polymerization [66]. The optimized hybrid electrode with 20 wt.% CNF delivered a specific capacitance of 421.5 F g^−1^, and the assembled all-solid-state supercapacitor maintained a superior electrochemical performance, even under repeatedly bending for 1000 cycles. In another work [67], carbon black (CB) was used to dope carbon nanofiber (CNF), and the PANI layer was electrosprayed on the flexible CB@CNF substrate (Figure 4a). The formed core-shell CB@CNF/PANI composite membrane was used as flexible electrodes, and delivered a specific capacitance of 501.6 F g^−1^ at 0.5 A g^−1^ in a three-electrode system. Moreover, the fabricated flexible and wearable symmetric device could be bent in various angles up to 180°. 

PS microspheres were also adopted to fabricate 3D PS/reduced graphene (PS/rGN) film by filtering the mixed suspension. As a flexible substrate, the PS/rGN film was used to grow PANI nanowires by in situ polymerization. After removing PS spheres, porous rGN/PANI composite film was obtained [70], and delivered a specific capacitance of 740 F g^−1^ or 581 F cm^−3^ at 0.5 A g^−1^. In addition, integrating exfoliated graphite (ExG), cellulose, PANI and silver nanoparticles (AgNPs), Khosrozadeh et al. [71] prepared a flexible PANI/ExG/cellulose/AgNPs hybrid film by vacuum filtration method. Through optimizing the ratio of different components, the hybrid film electrode achieved a high capacitance of 240.10 F g^−1^ or 3.84 F cm^−2^ at a discharge current of 5 mA.

Besides filtration method, a flexible graphene paper can be prepared by depositing the graphene suspension on smooth Teflon substrate. As a working electrode, the PANI nanorods were electropolymerized on the peeled reduced GO paper (Figure 4b) [68]. The flexible free-standing graphene-PANI paper electrode delivered a capacitance of 763 F g^−1^ at 1 A g^−1^, which was much higher than that of pure graphene paper and PANI film. In addition, flexible graphene woven fabric (GWF) and graphite sheet (FGS) were also selected as substrates for growing PANI. For example, Zang et al. [69] grew PANI on the flexible GWF by electropolymerization method, and prepared GWF+PANI composite electrode for flexible solid-state supercapacitors (Figure 4c). The specific capacitance of PANI coated GWF increased to 23 mF cm^−2^ from 2 mF cm^−2^ of the original GWF electrode, with no sacrifice of the flexibility, and the capacitance retained ~100% after 2000 cycles. Xin et al. [72] prepared self-supporting graphene (SSG) on a flexible FGS, and further electrodeposited PANI thin film on FGS-SSG. The flexible FGS-SSG/PANI composite electrodes exhibited a specific capacitance of 491.3 F g^−1^ based on PANI. Lin et al. [73] deposited PANI layer on the conductive carbon woven fabric (CWF), and then wrapped another rGO layers through the electrostatic interaction. The prepared PANI/rGO fabric composite had good flexibility and conductivity, which exhibited a capacitance of 790 F cm^−2^ at 1 A cm^−2^, and the capacitance retained 88.9% after cycling for 5000 cycles.

### 4.2. Graphene Framework Based Composites

Compared to other 3D carbon frameworks, graphene frameworks have an abundant interconnected porous structure, high specific surface area and excellent conductivity, which can be used to grow a great many of PANI nanoparticles. In this section, we introduce the advance of 3D graphene/PANI composites according to the preparation methods of 3D graphene framework, including chemical vapor deposition (CVD), porous template method, hydrothermal treatment, freeze-drying method and self-assembly of graphene. 

#### 4.2.1. Chemical Vapor Deposition (CVD)

**Figure 5:**
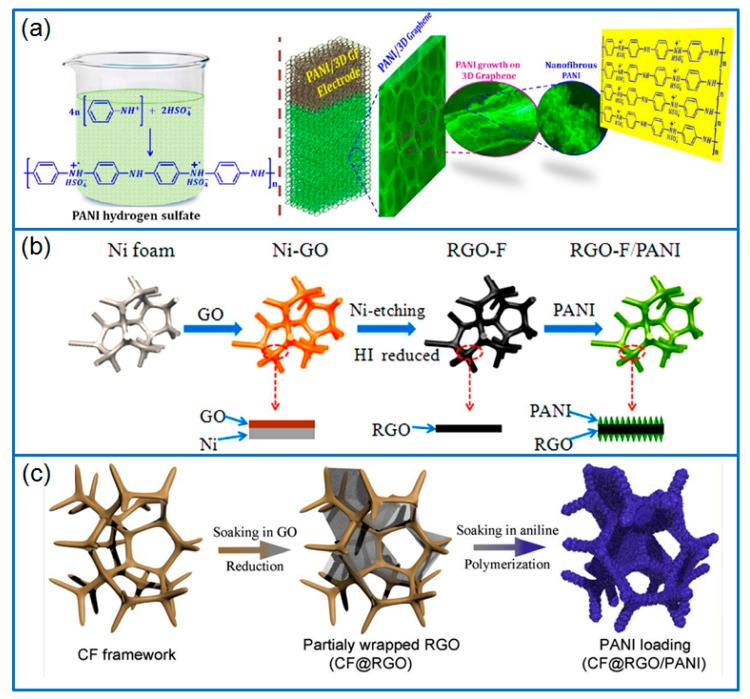
(**a**) Schematic for chemically growing PANI on 3D graphene framework; produced with permission [76]. Copyright 2014, Royal Society of Chemistry. (**b**) Preparation of rGO-F and rGO-F/PANI composites by dip coating method; produced with permission [79]. Copyright 2014, Royal Society of Chemistry. (**c**) Schematic illustration of the preparation of CF@rGO/PANI ternary composite; produced with permission [80]. Copyright 2018, Elsevier.

CVD is a popular way to prepare 3D graphene framework [74]. Graphene sheets are deposited on nickel foam (NF) substrate by heating the carbon source in tube furnaces, after removing the NF, 3D macroporous graphene network is prepared. By using the graphene framework, Yu et al. [75] grew vertically aligned PANI nanocones on graphene by template-free electrodeposition. The composite electrodes exhibited a specific capacitance of 751.3 F g^−1^ in 1 M HClO_4_, with 93.2% capacitance retention after 1000 cycles. Furthermore, 3D graphene network derived from CVD method was served as porous conducting network for growing PANI nanofibers in H_2_SO_4_ solution (Figure 5a) [76]. In a three-electrode system, the freestanding composite electrode exhibited a capacitance of 1002 F g^−1^ at 1 mA cm^−2^. In another work [77], PANI nanofibers (PANI-NFS) were electrodeposited on the working electrode of graphene foam (GF). The pore diameters of 3D hierarchical porous PANI-NFS/GF composite could be adjusted by changing electrodeposition condition. When the GF filling factor was 11%, the composite electrode had the best performance, with a capacitance of 1474 F g^−1^ (86 F cm^−3^) at 0.47 A g^−1^, and the capacitance retention was 83% after charging/discharging for 15,000 cycles. The shape of NF substrate is also important for the feature of graphene framework. For example, a wavy-shaped NF substrate can be used for wavy-shaped graphene framework [78]. After electrodeposited PANI film on the wavy substrate and attached it in elastomeric substrates, the wavy-shaped graphene/PANI electrodes could be stretched and bent easily. In a stretchable all solid-state supercapacitor, the wavy-shaped electrodes delivered a specific capacitance of 261 F g^−1^, and capacitance retention of 89% for 1000 cycles. 

#### 4.2.2. Porous Template Method

Besides CVD method, porous NF can be served as a porous template to prepare 3D porous rGO foam (rGO-F) by repeatedly dipping and drying. With this way, Yu et al. [79] prepared porous rGO-F and grew PANI nanowires on the foam by in situ polymerization (Figure 5b). When assembled in a symmetric supercapacitor, the prepared rGO-F/PANI composites displayed a specific capacitance of 790 F g^−1^ or 205.4 F cm^−3^. Furthermore, the capacitance of this device retained 80% after 5000 cycles. Except for dipping and drying method, Wu et al. [81] prepared 3D graphene foam (GF) on NF by a roll-forming method, then the PANI was electrochemically deposited on the GF to prepare freestanding PANI/HGNF composite electrode. In another work [82], a “templating and embossing” method was developed to prepare hierarchical porous 3D graphene skeletons. At first, NF was soaked in the mixed suspension of modified PS spheres and GO. The PS spheres served as “spacers” and prevented the stack of rGO sheets. After removing NF and heat annealing in N_2_, hierarchical porous 3D frGO-F skeleton was prepared and in situ polymerized PANI nanowires. The flexible frGO-F/PANI composites were obtained with macro/mesoporous structure. Attributed to the synergistic effect of 3D bicontinuous hierarchical porous framework and PANI nanowires, as-assembled symmetric supercapacitor delivered a specific capacitance of 939 F g^−1^, and high capacitance retention of 88.7% for 5000 cycles. Besides porous NF, carbon foam was also used as 3D freestanding porous template. In Hong’s work [80], the carbon foam filled with graphene nanosheets was employed to grow PANI nanofibers (Figure 5c), and the addition of graphene sheets into carbon foam framework increased the loading amount of PANI. As a result, the ternary composite consisted of carbon foam, graphene and PANI had a high PANI loading of 85.5 wt.%. When assembled in symmetrical supercapacitors, the optimized ternary composite electrodes exhibited a capacitance of 868.5 F g^−1^ at 1 A g^−1^, and the PANI capacitance reached 1003 F g^−1^.

#### 4.2.3. Hydrothermal Method 

Hydrothermal method is a simple way to prepare 3D porous graphene hydrogels. Under the high pressure water vapor, the self-assembly of graphene nanosheets occurred with the removal of oxygen-containing groups of GO, and the π-π interaction of hydrophobic rGO sheets induced the formation of macroporous structure. In recent years, the hydrothermal method has been widely adopted to fabricate 3D graphene hydrogels (GH) with highly conductive, mechanical robust and macroporous structure [83]. Based on the prepared GH framework, PANI thin layer was conformally coated on the graphene sheets by electrodeposition [84]. As-prepared binder-free GH/PANI electrode showed a high specific capacitance of 710 F g^−1^ at 2 A g^−1^, and an excellent rate capability of 73% capacitance retention at 100 A g^−1^. Zhao et al. [85] also prepared 3D graphene hydrogels by hydrothermal reaction, then generated PANI nanorods on the graphene surface with in situ polymerization. The 3D graphene/PANI composite electrode exhibited a capacitance of 352 F g^−1^ at 10 mV s^−1^. Moreover, based on the highly concentrated GO/PANI gels casted on the PTFE tape, 3D porous rGO/PANI hybrid film was prepared through steamed water regulation after hydrothermally treating at 200 °C for 5 h [86]. When used in a three-electrode system, the rGO/PANI film delivered a capacitance of 1182 F g^−1^ at 1 A g^−1^. Meanwhile, the film electrodes also had a high capacitance of 808 F g^−1^ and an energy density of 28.06 Wh kg^−1^ in a symmetric supercapacitor.

**Figure 6:**
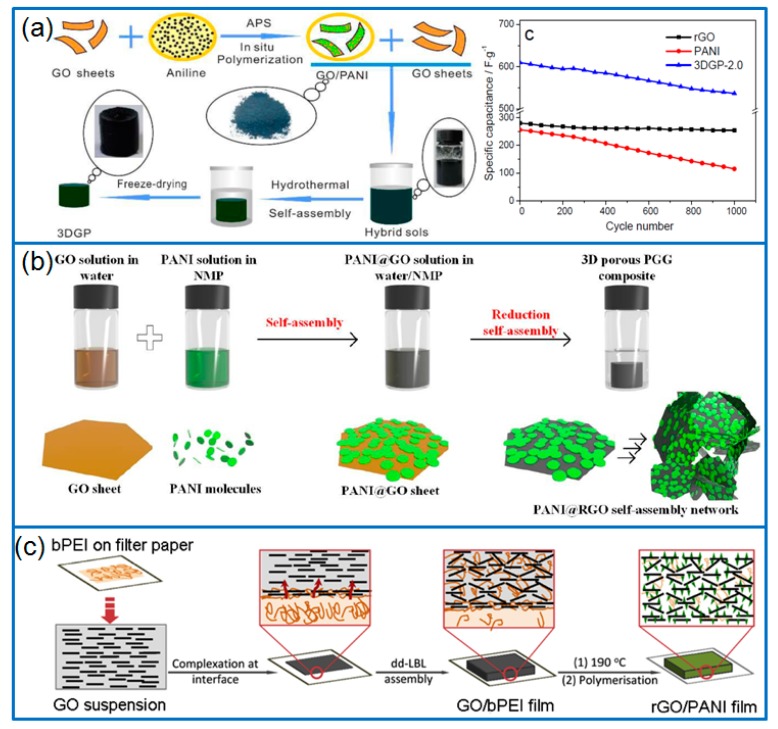
(**a**) Schematic for preparing PANI and 3DGP, and the cycling performance of different electrodes; produced with permission [88]. Copyright 2015, Royal Society of Chemistry. (**b**) Preparation of PANI@rGO network by self-assembly method; produced with permission [95]. Copyright 2018, Royal Society of Chemistry. (**c**) Schematic illustration for preparing rGO/PANI composite film by diffusion driven layer-by-layer assembly; produced with permission [96]. Copyright 2017, Elsevier.

Commonly speaking, a two-step procedure is required to prepare 3D graphene/PANI composites through hydrothermal method. The first step is the preparation of graphene hydrogels, and the second step is the synthesis of PANI on the hydrogel. However, in order to effectively avoid the restacking of rGO sheets, PANI can be grown on the graphene sheets firstly. For example, Wang et al. [87] prepared 3D graphene/PANI (3DGP) hydrogels by a modified two-step method (Figure 6a). In the first process, the PANI was grown on GO sheets by chemical oxidative method, then the mixed suspension of GO and PANI/GO composite was hydrothermally treated. The 3DGP composite had a specific capacity of 648 F g^−1^ at 0.5 A g^−1^ in a three-electrode cell. In another work, the PANI was grown on rGO firstly by interfacial polymerization. Then, through a reassembly of as-prepared GO@PANI and GO sheets in a hydrothermal reaction, 3D hierarchical porous rGO-PANI aerogels were fabricated [88]. Due to the high specific surface area (337 m^2^ g^−1^), high conductivity of graphene framework and the sandwiched PANI/graphene/PANI layered structure, the hybrid aerogel electrodes delivered a high areal capacitance of 453 mF cm^−2^ in symmetric supercapacitors. Hoa et al. [89] prepared PANI grafted rGO aerogel composite (rGO-g-PANI) with the two-step method. The GO-g-PANI was synthesized firstly with the chemical oxidative method under the presence of GO-NH_2_. In the second step, a hydrothermal treatment was conducted to prepare rGO-g-PANI aerogels. After coated on the glassy carbon electrode, the composite exhibited a high specific capacitance of 1600 F g^−1^ at 12 A g^−1^ and capacitance retention of 91.3% for 3000 cycles.

In order to explore the influence of hydrothermal conditions on the PANI morphologies and electrochemical performance, Wang et al. [90] synthesized PANI nanostructures on the GO sheets, and hydrothermally treated the GO/PANI suspension under different temperatures. Results showed that sheet-like graphene/PANI composite synthesized at 120 °C had a high specific capacitance of 532.3 F g^−1^ at 2 mV s^−1^. Except for hydrothermal temperature, the polymerization temperature of aniline monomers also affected the electrochemical performance of graphene/PANI composites. Ates et al. [91] prepared graphene hydrogels (GH) and grew PANI on this porous framework, and the influence of polymerization temperatures on the electrochemical performance of GH/PANI composite was discussed. The GH/PANI composite electrodes synthesized at 25 °C exhibited a specific capacitance of 2.78 F cm^−2^ (or 323.9 F g^−1^) in symmetric supercapacitors, which was much higher than the composite synthesized at 0 °C. In order to increase the active sites on graphene nanosheets to grow more PANI, GO sheets were chemically treated in HNO_3_ and H_2_O_2_, and then hydrothermally treated to prepare 3D multi-growth site graphene (MSG) foam [92]. Compared to conventional compacted graphene foam, the MSG foam had a sloppy structure, which facilitated the ion diffusion. After growing PANI on the MSG foam by in situ polymerization, the capacitance of 3D MSG/PANI composite enhanced to 912 F g^−1^ from 432 F g^−1^, and the capacitance retention rate was 89.5% after 10,000 cycles at 10 A g^−1^.

Nitrogen doped graphene framework can also be synthesized by hydrothermal reaction in NH_3_·H_2_O solution. Zhu et al. [93] prepared 3D porous nitrogen-doped graphene/PANI (N-GE/PANI) foam with nitrogen-doped graphene sheets encapsulating PANI spheres. The N-GE/PANI composite foam showed a high specific capacitance of 528 F g^−1^ (0.1 A g^−1^), and a high capacitance retention rate of 95.9% for 5000 cycles. Similar to NH_3_·H_2_O, the urea was also employed as both doping nitrogen source and reducing agent. After one-step hydrothermal treatment, 3D nitrogen-doped graphene/PANI hydrogels (RGNP) were obtained. The RGNP electrode exhibited a specific capacitance of 589.3 F g^−1^ at 3 mA cm^−2^ in a three-electrode cell. In addition, by using glucose as the reducing agent and CaCO_3_ particles as templates, 3D-rGO porous framework was obtained by a hydrothermal reaction [94], then CaCO_3_ particles were removed in HCl solution, and PANI nanowires were grown on 3D-rGO framework by in situ polymerization. The prepared 3D-rGO/PANI composite electrode delivered a capacitance of 243 F g^−1^ at 1 A g^−1^.

#### 4.2.4. Freeze-Drying Method

Different from hydrothermal method, freeze-drying method is much easier, with no hydrothermal process. Liu et al. [97] adopted PS spheres as sacrificial template and melamine as the nitrogen source, and prepared nitrogen-doped 3D interconnected graphene (N-3D-rGO) frameworks by freeze-drying and sintering. Based on this framework, PANI nanowires (PANI-B) were generated using APS and β-MnO_2_ as the oxidants. As-prepared N-3D-rGO/PANI-B had a capacitance of 282 F g^−1^ at 1 A g^−1^, this performance was much better than that of the composite prepared by using only APS oxidant. During the freezing process, the control of the temperature gradient also affected the performance of graphene aerogel. Wu et al. [98] prepared unidirectional graphene aerogel (UGA) by unidirectionally freezing GO suspension with temperature gradient. After growing PANI nanowires, the freestanding porous UGA/PANI composites were fabricated. Compared with conventional hydrogels, the long-range ordered interconnected porous structure of UGA facilitated the ion diffusion. The UGA/PANI composite electrode exhibited a specific capacitance of 538 F g^−1^ (1 A g^−1^), much higher than that of conventional graphene aerogel/PANI composite (345 F g^−1^).

#### 4.2.5. Self-Assembly of Graphene

Through heating the mixture of GO and in-situ polymerized GO/PANI in sodium ascorbate solution at 95 °C, Li et al. [99] prepared 3D graphene (3D-G)/PANI composite, and pressed the composite on the prestretched elastic substrates for stretchable supercapacitors. The 3D-G/PANI electrode exhibited a capacitance of 567 F g^−1^ at 1 A g^−1^. However, in stretchable solid-state asymmetric supercapacitors consisted of 3D-G/PANI and 3D-G, the energy density reached 77.8 Wh Kg^−1^, and the capacitance retention was 95.6% for 10,000 cycles, moreover, the capacitance retained 91.2% after stretching for 100 cycles. In another work, Li et al. [100] synthesized graphene/PANI composite nanosheets by using interfacial polymerization, and embedded them into graphene framework under a heating treatment at 95 °C. As-prepared 3D-G/PANI composite was fabricated into a flexible conductive film for all-solid-state supercapacitor, and exhibited a high specific capacitance of 665 F g^−1^ (847 F cm^−3^), with a stable cycling for 10,000 cycles.

By using a self-assembly strategy, a kind of self-suspended polyaniline (S-PANI) containing PEG segment [27] was used for fabricating 3D rGO/S-PANI aerogel [101]. A cross-linking reaction occurred between GO sheets and functionalized S-PANI through the π-π interactions or forming hydrogen bonding, then the GO was reduced later with hydrothermal treatment. The freestanding rGO/S-PANI composite delivered a specific capacitance of 480 F g^−1^ at 1 A g^−1^, and the capacitance retained 96.14% after 10,000 cycles at 10 A g^−1^. In addition, Wu et al. [95] prepared 3D porous PANI/rGO composite gels with molecular-level uniformity by using a two-step self-assembly method (Figure 6b). In the first step, the PANI particles were assembled on the GO sheets in water/*N*-methyl-2-pyrrolidone and formed PANI@rGO nanosheets. In the second assembly, the reduction process of GO at 90 °C induced the 3D assembly of PANI@GO nanosheets, and PANI coated rGO framework was obtained. The composite electrode exhibited a high specific capacitance of 808 F g^−1^ (53.33 A g^−1^) or 5.717 F cm^−2^ at 377.4 mA cm^−2^.

By using a electrostatic layer-by-layer (LbL) assembly method and electropolymerization, Gupta et al. [102] prepared PANI/rGO multilayer, and discussed the effect of rGO loading on their electrochemical performance. Different from traditional assembly of graphene sheets, a diffusion driven layer-by-layer (dd-LbL) assembly was developed to prepare 3D porous graphene foam [103,104], in which, the positively charged branched polyethyleneimine (bPEI) was used to complex with GO sheets, and 3D GO/bPEI hydrogels were formed with GO sheets stacked layer by layer. By this dd-LbL assembly method, Hong et al. [96] prepared 3D rGO framework as a template and grew PANI nanoparticles through in situ polymerization (Figure 6c). As freestanding electrodes for a sandwiched supercapacitor, the rGO/PANI composite showed a specific capacitance of 438.8 F g^−1^ at 0.5 A g^−1^, and the capacitance of PANI nanoparticles was calculated as 763 F g^−1^.

### 4.3. Printable Composites 

With the rapid development of printing techniques [105], some electrodes can be directly printed with desired shapes and complicated microstructures [106]. However, the performance of printable supercapacitors is mainly dependent on the ink materials. In this section, some PANI based inks and corresponding printing techniques are summarized. 

**Figure 7:**
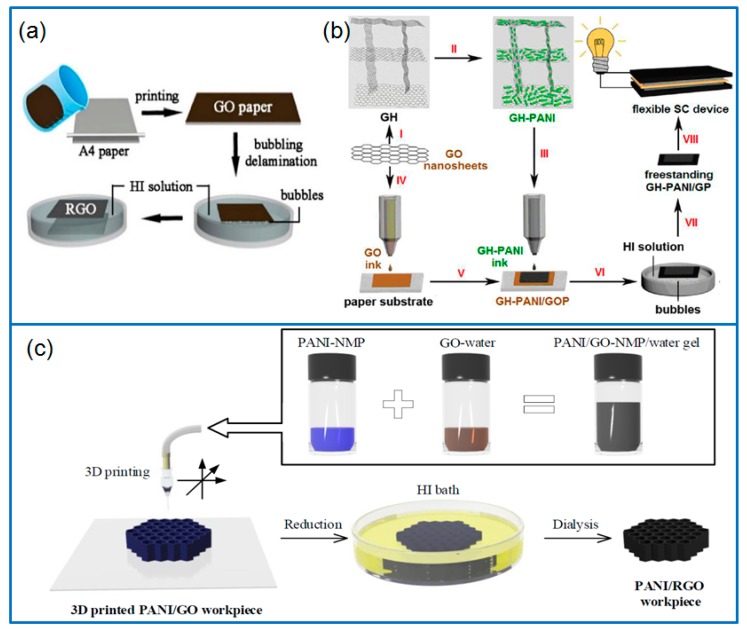
(**a**) Schematic for preparing a freestanding rGO paper; produced with permission [107]. Copyright 2015, Nature Publishing Group. (**b**) Preparation of GH-PANI/GP composite for flexible devices; produced with permission [108]. Copyright 2014, American Chemical Society. (**c**) Schematic illustration for preparing PANI/rGO composite by 3D printing; produced with permission [109]. Copyright 2018, American Chemical Society.

By using a reproducible printing method and bubbling delamination, a flexible freestanding rGO paper (Figure 7a) was prepared with light-weight, highly conductive and mechanically robust [107]. The rGO paper was then covered with PANI nanofibers by in situ electropolymerization. After deposited another rGO layer on the rGO/PANI by dip-coating and reduction, the flexible sandwiched rGO/PANI/rGO hybrid paper was obtained and showed an enhanced electrochemical performance than that of rGO/PANI composite, with a capacitance of 581 F g^−1^ (1 A g^−1^) and a capacitance retention of 85% after 10,000 cycles. Chi et al. [108] prepared a freestanding graphene paper (GP) as flexible substrate by an inkjet printing method (Figure 7b). Meanwhile, 3D porous graphene hydrogel (GH) was adopted to grow PANI by chemical oxidative method. After ball milling the PANI based composite and dispersing the powder in solvent, GH-PANI inks were prepared. Through overprinting GH-PANI inks on the GP, a freestanding GP supported GH-PANI composite (GH-PANI/GP) was finally prepared. As flexible electrodes for all-solid-state symmetric supercapacitors, the GH-PANI/GP composite delivered a specific capacitance of 864 F g^−1^ (1 A g^−1^) or 190.6 mF cm^−2^ (0.5 mA cm^−2^), and the energy density was 24.02 Wh kg^−1^ at 400.33 W kg^−1^. In addition, Wang et al. [109] developed a direct ink writing (DIW) to fabricate PANI-based electrodes. The PANI/GO gel inks was crucial for the 3D printing technique, which were fabricated by the self-assembly of GO and PANI in a mixed solvent of water and NMP. After reducing the printed composites in HI (hydroiodic acid) solution, freestanding interdigital PANI/rGO electrodes were obtained (Figure 7c). The printed planar solid-state supercapacitor exhibited a specific capacitance of 1329 mF cm^−2^. 

Different from traditional 3D printing techniques, Song et al. [110] developed a solution- processible method to prepare microelectrode patterns. The crucial active material was an aqueous solution of graphene/sulfonated PANI (rG/SP), which ensured the formation of interdigital patterns after plasma etching. The micro-supercapacitors with rG/SP electrodes delivered a high capacitance of 3.31 mF cm^−2^ (16.55 F cm^−3^). Moreover, this micro-device also showed a stable capacitance after bending and twisting tests, which was suitable for wearable and portable electronic devices.

When compared to 2D graphene/PANI, 3D graphene/PANI composites can be directly served as electrodes for their freestanding feature, especially in the two-electrode supercapacitors. Moreover, 3D graphene/PANI composites exhibit a superior specific capacitance and cycling performance, as given in Table S1. Therefore, developing high performance 3D graphene/PANI composites will promote the rapid development of flexible wearable supercapacitors, high performance binder-free supercapacitors and printable supercapacitors.

## 5. Summary and Perspective

### 5.1. Summary

The hybrid electrodes of graphene and PANI have been widely reported in high performance supercapacitors. In this review, we summarized the progress of synthesis methods of PANI and graphene/PANI composites, and their application in supercapacitors. 

**Figure 8:**
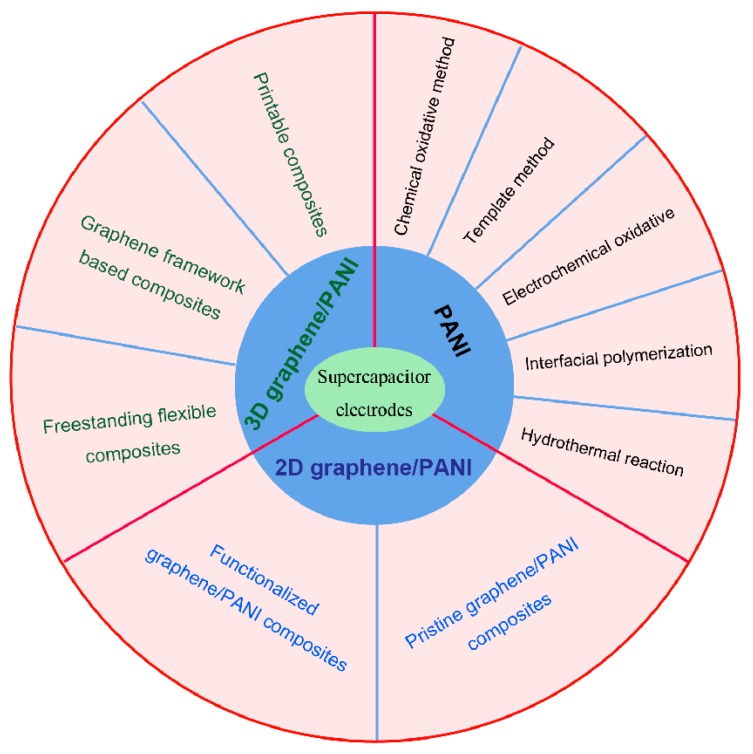
Schematic for preparing PANI, 2D and 3D graphene/PANI composites for supercapacitor electrodes.

As shown in Figure 8, five synthesis methods of PANI were introduced in detail, which included the chemical oxidative method, template method, electrochemical oxidative method, interfacial polymerization and hydrothermal reaction. Among these methods, there were more than 80% works involving chemical oxidative method and electrochemical method. In the preparation of 2D graphene/PANI composites, functionalized graphene with amine groups were adopted for preparing covalently-grafted PANI/GO composites by initiating the polymerization of aniline. Compared to the decorated graphene by hydrogen bonding and p-p stacking interactions, chemical grafting strategy was more effective for enhancing the capacitance and cycling stability of graphene/PANI composites. Compared to 2D graphene/PANI composites, the design of 3D graphene frameworks was a basis for preparing 3D graphene/PANI composites. In that section, we mainly introduced the preparation methods of graphene framework, including chemical vapor deposition, porous template method, hydrothermal treatment, freeze-drying method and self-assembly of graphene. Based on these porous graphene templates, 3D freestanding graphene/PANI composites were prepared, and exhibited an excellent rate capability and long-term cycling stability. In addition, we also summarized the advance of flexible graphene/PANI composites and printable graphene/PANI composites. In printable composites, the developing of PANI based inks determined the printing methods and the performance of supercapacitors. Furthermore, the printing techniques achieved the fast fabrication of complicated shape electrodes, which promote the rapid development of portable and wearable electronic devices. We also summarized the specific capacitance and cycling performance of various graphene/PANI composites in Table S1. Among 59 papers on composite electrodes, 22% works (13 papers) had a specific capacitance more than 800 F g^−1^, and only 6.78% works (four papers) achieved the higher capacitance (≥800 F g^−1^) in two-electrode systems. From the long-term cycling performance, 16.95% works (10 papers) listed the capacitance retention over 10,000 cycles, and only 10.17% works (six papers) had a high capacitance retention (≥85%). Therefore, there is still a long way to further enhance the electrochemical performance of graphene/PANI composite electrodes.

### 5.2. Perspective

A great progress has been achieved in the field of PANI and graphene/PANI composites, and the specific capacitance and long-term cycling performance of graphene/PANI electrodes have enhanced greatly. However, there are still some challenges with graphene/PANI composite electrodes, such as, the controllable synthesis of PANI nanostructures, the agglomeration of PANI particles and graphene nanosheets, the optimized ratio of graphene and PANI, high cost of graphene and the design of high performance graphene frameworks. These challenges are just retarding the rapid development and the commercialization of graphene/PANI electrodes in supercapacitors. In order to solve these problems, we put forward some strategies to develop graphene/PANI composites for high performance supercapacitors.

(1) Selecting suitable synthesis method of PANI and considering the combination of chemical synthesis and surface treatment. According to the features of graphene, a suitable synthesis method should be adopted firstly. For example, chemical oxidative method and interfacial polymerization are fit for growing PANI nanostructures on the well dispersed graphene suspension or porous graphene frameworks; while electrochemical deposition method are suitable for growing PANI on the flexible graphene film. In addition, the surface treatment of PANI in chloroform could greatly enhance the specific surface area and pore volume of PANI, which improve the rate capability of PANI. Therefore, the positive effect of surface treatment on PANI should not be ignored. 

(2) Improving the cost performance of graphene/PANI composites based on functionalized graphene. Compared to the simple hybridization, covalently-grafted graphene can initiate the polymerization of aniline monomers. The functionalized graphene/PANI composites exhibit a superior electrochemical performance than that of ordinary graphene/PANI composites. In addition, the controllable synthesis of PANI is really crucial for improving the cost performance, for example, synthesizing PANI by adjusting polymerization temperature, adopting compound oxidants, or selecting suitable dopants. 

(3) Designing hierarchical porous graphene framework and hybridizing with an optimized loading of PANI nanostructures. Freestanding porous graphene framework overcomes the blocking effect of polymer binders and ensures an excellent conductivity; moreover, it also facilitates the rapid transfer of ions, which greatly improve the rate capability of supercapacitors. In addition, based on the graphene framework, the PANI nanostructures and their loading amount should be controlled to achieve the high performance supercapacitors.

(4) Developing suitable graphene/PANI composite inks and corresponding printing techniques. In recent years, printing technique has been developed to fabricate complicated electrodes repeatedly and quickly. However, the inherent feature of inks will decide the performance of printed supercapacitors. Therefore, high performance graphene/PANI inks can be prepared by adjusting their mass ratio, conductivity, flowability and mechanical strength. Printable inks and suitable printing techniques will promote the fast development of intelligent energy storage microdevices and flexible wearable supercapacitors. 

## Supplementary Materials

The following are available online at https://www.mdpi.com/1996-1944/12/9/1451/s1, Table S1: The performance of graphene and PANI composites for supercapacitors.
