# Supplementary material for: Recent Progress on Graphene/Polyaniline Composites for High-performance Supercapacitors
Source: Materials (Basel). 2019 May 5;12(9):1451. doi: 10.3390/ma12091451 (PMC6540261; doi:10.3390/ma12091451)
Supplement: Supplementary file 1 [file materials-12-01451-s001.pdf]

Supplementary material

Review

Recent Progress on Graphene/Polyaniline Composites for High-performance Supercapacitors

Xiaodong Hong *, Jiawei Fu, Yue Liu, Shanggong Li, Xiaoliang Wang, Wei Dong and Shaobin Yang

College of Materials Science and Engineering, Liaoning Technical University, Fuxin 123000, China; fjw1518816615@163.com (J.F.); liuyue471804@163.com (Y.L.); hxdhit@163.com (S.L.); ningke@163.com (X.W.); lgddongwei@163.com (W.D.); yunwen2004@126.com (S.Y.)

* Correspondence: hongxiaodong@lntu.edu.cn; Tel.: +86-1384-187-7730

Abbreviations

polyaniline (PANI)
two dimensional (2D)
three dimensional (3D)
ammonium persulfate (APS)
graphene oxide (GO)
vanadium pentoxide hydrate ($V_2O_5{\cdot}nH_2O$)
p-toluenesulfonic acid (PTSA)
polystyrene (PS)
polymethyl methacrylate (PMMA)
reduced graphene oxide (rGO)
mesoporous silica ($mSiO_2$)
PANI hollow fibers (PANI-HF)
polyacrylonitrile (PAN)
multidimensional (MD)
polyvinyl alcohol (PVA)
polyethylene glycol (PEG)
N-methylpyrrolidinone (NMP)
polycarbonate (PC)
dodecyl benzene sulfonic acid (DBSA)
porous tubular carbon (PTC)
glassy carbon electrode (GCE)
stainless steel fabric (SSF)
chloroform ($CHCl_3$)
$CHCl_3$-treated PANI (HSSA-PANI)
pristine graphene (PG)
graphene quantum dots (GQDs)
graphene microspheres (GMS)
sulfonated graphene (GS)
aminated graphene (GN)
amino-functionalized graphene sheets (AFG)
graphene (G)
p-phenylenediamine (PPD)
PANI nanofiber/N-doped graphene hydrogels (PNGH)
graphene aerogel (GA)
m-phenylenediamine (mPD)
graphene hydrogel (PGH)
phytic acid (PA)
amino-triazine (AT) functionalized rGO (ATrGO)
2, 4, 6-Trichloro-[1, 3, 5] triazine (TCTA)
ST functionalized graphene sheets (STGNS)
tannic acid (TA)
pyrenebutyric acid (PBA)
oriented graphene hydrogel (OGH)
cellulose nanofibers (CNF)
graphite nanoplatelets (GNP)
carbon black (CB)
carbon nanofiber (CNF)
PS/reduced graphene (PS/rGN)
silver nanoparticles (AgNPs)
exfoliated graphite (ExG)
graphene woven fabric (GWF)
graphite sheet (FGS)
carbon woven fabric (CWF)
chemical vapor deposition (CVD)
nickel foam (NF)
rGO foam (rGO-F)
multi-growth site graphene (MSG)
nitrogen-doped graphene/PANI (N-GE/PANI)
nitrogen-doped 3D interconnected graphene (N-3D-rGO)
unidirectional graphene aerogel (UGA)
self-suspended polyaniline (S-PANI)
graphene paper (GP)
graphene/sulfonated PANI (rG/SP)

Table S1. The performance of graphene and PANI composites for supercapacitors.

Electrode materials	C_m (F g^{-1})	C_A (mF cm^{-2})	C_V (F cm^{-3})	Cycle life	Refer
graphene/PANI electrode	480 (0.1 A g^{-1}) 3-electrode				38
rGO-PANI electrode	286 (5 mV s^{-1}) 3-electrode			94% capacitance after 2000 cycles (50 mV s^{-1})	39
GO/PG/PANI ternary composite	793.7 (1 A g^{-1}) 3-electrode			80% capacitance after 1000 cycles (100 mV s^{-1})	40
GQDs-PANI composites	1044 (1 A g^{-1}) 3-electrode			80.1% capacitance after 3000 cycles (1A g^{-1})	42
Microspherical polyaniline/graphene (PANI/GMS)	338 (20 mV s^{-1}) 2-electrode			87.4% capacitance after 10000 cycles (3A g^{-1})	43
PANI/G-MS	596.2 (0.5 A g^{-1}) 447.5 (20 A g^{-1}) 3-electrode			83.7% capacitance after 1500 cycles (2A g^{-1})	44
PANI/GO composite	475 (5 A g^{-1}) 3-electrode			90% capacitance after 2000 cycles (10A g^{-1})	45
PANI-GS composite	863.2 (0.2 A g^{-1}) 3-electrode			85.6% capacitance after 1000 cycles (1A g^{-1})	46
GMPH7 electrode	514.3 (1 A g^{-1}) 3-electrode	584.7 2-electrode		12.9% capacitance loss after 1000 cycles (10A g^{-1})	47
DN-PGH/PANIPA		3488.3 (0.5 mA cm^{-2}) 2-electrode	872	82.1% capacitance after 10000 cycles (20 mA cm^{-2})	48
Polyaniline-graphene composites	1295 (1 A g^{-1}) 3-electrode			98% capacitance after 1500 cycles(100 mV s^{-1})	51
PANI/GO	442 (1 A g^{-1}) 3-electrode			83% capacitance after 2000 cycles (2A g^{-1})	52
Chemically grafted graphene-polyaniline composite (a-G-PANI200)	422 (1 A g^{-1}) 3-electrode			68.7% capacitance after 1000 cycles (1A g^{-1})	53
PANI nanofiber/N-doped graphene hydrogels (PNGH)	610 (1 A g^{-1}) 3-electrode			94.3% capacitance after 1000 cycles (20A g^{-1})	54
GA/PANI composite	108 (1 A g^{-1}) 3-electrode			83.2% capacitance after 10000 cycles (10A g^{-1})	55
PANI-ATRGO	1510 (1 A g^{-1}) 3-electrode			89% capacitance after 1500 cycles(100 mV s^{-1})	59

sulfur functionalized PANI/FrGO composite films	692 (1 A g^{-1}) 3-electrode 324.4 (1 A g^{-1}) 2-electrode			83.3% capacitance after 1000 cycles (10A g^{-1})	60
PANI-STGNS10	1225 (1 A g^{-1}) 3-electrode			85.7% capacitance after 1500 cycles (100 mV s^{-1})	61
Flexible rGO/PANI nanocomposite film		920 2-electrode	1314.3	80% capacitance after 2000 cycles (7 mA/cm^2)	62
Porous PANI-RGO composite	630 (0.5 A g^{-1}) 3-electrode			81% capacitance after 5000 cycles	63
Flexible paper-like film of PANI nanofibers (PANI-NFs) and chemically converted graphene (CCG)	210 (0.3 A g^{-1}) 2-electrode			21% loss after 800 cycles (3A g^{-1})	64
OGH-PANI composite film	530 (0.5A g^{-1}) 2-electrode			80% capacitance after 10000 cycles (10A g^{-1})	65
Core-shell structured CB@CNF/PANI membrane	501.6 (0.5 A g^{-1}) 3-electrode			91% capacitance after 5000 cycles	66
PANI nanorods on the peeled reduced GO paper	763 (1 A g^{-1}) 3-electrode			82% capacitance after 1000 cycles (5A g^{-1})	67
GWF+PANI composite electrode		23 2-electrode		~100% capacitance after 2000 cycles	68
CNF/GNP substrate generated with worm-like PANI nanorods	421.5 (1 A g^{-1}) 2-electrode			78.3% capacitance after 1000 cycles (8A g^{-1})	69
Porous rGN/PANI composite film	740 (0.5 A g^{-1}) 3-electrode		510	87% capacitance after 1000 cycles (10A g^{-1})	70
PANI/ExG/cellulose/AgNPs	240.10	3840 at 5 mA			71
flexible FGS-SSG/PANI composite electrodes	491.3 (50 mV s^{-1}) 3-electrode			86% capacitance after 3000 cycles (50 mV s^{-1})	72
PANI/rGO fabric composite		790 F cm^{-2} (1 A cm^{-2}) 3-electrode		88.9% capacitance after 5000 cycles (10A g^{-1})	73
3D graphene framework grew vertically aligned PANI nanocones	751.3 (1 A g^{-1}) 3-electrode	3.0		93.2% capacitance after 1000 cycles (10A g^{-1})	75
3D graphene network growing PANI nanofibers	1002 (1 mA cm^{-2}) 3-electrode			86.5% capacitance after 5000 cycles (4 mA/cm^2)	76
3D hierarchical porous PANI-NFS/GF composite	1474 (0.47 A g^{-1}) 3-electrode		86	83% after 15,000 cycles	77

Graphene/PANI electrodes	261.24 (0.38 A g^{-1}) 2-electrode			89% capacitance after 1000 cycles (1mA/cm^2)	78
RGO-F/PANI	790 (1 A g^{-1}) 2-electrode		205.4	80% capacitance after 5000 cycles (1 A g^{-1})	79
Carbon foam@reduced graphene oxide scaffold grown with PANI nanofibers	868.5 (1 A g^{-1}) 2-electrode			94.1% capacitance after 2000 cycles (10A g^{-1})	81
Flexible fRGO-F/PANI composites	939 (1 A g^{-1}) 2-electrode			88.7% capacitance after 5000 cycles (1 A g^{-1})	82
GH/PANI electrode	710 (2 A g^{-1}) 2-electrode			86% capacitance after 1000 cycles (2A g^{-1})	84
3-D graphene/PANI	352 (10 mV s^{-1}). 2-electrode				85
3D hierarchical porous rGO-PANI aerogels		453		90% capacitance after 3000 cycles 100 mV s^{-1}	86
rGO/PANI (50%)	1182 (1 A g^{-1}) 3-electrode 808 (1A g^{-1}) 2-electrode			108% capacitance after 10000 cycles (10 A g^{-1})	87
3D graphene/PANI hydrogels	648 (0.5 A g^{-1}) 3-electrode			88% capacitance after 1000 cycles (1 A g^{-1})	88
3D RGO-g-PANI	1600 (12 A g^{-1}) 3-electrode			91.3% capacitance after 3000 cycles (4 A g^{-1})	89
Sheet-like graphene/PANI composite	532.3 (2 mV s^{-1}) 3-electrode			99.6% capacitance after 1000 cycles (50 mV s^{-1})	90
GH/PANI composite	323.9 (2 mV s^{-1}) 2-electrode			99% capacitance after 1000 cycles	91
3D MSG/PANI composite	912 (1 A g^{-1}) 3-electrode			86.4% capacitance after 10000 cycles (20A g^{-1})	92
(N-GE/PANI)	528 (0.1 A g^{-1}) 3-electrode			95.9% capacitance after 5000 cycles (5 A g^{-1})	93
3D-rGO/PANI composite electrode	243 (1 A g^{-1}) 3-electrode			86% capacitance after 1000 cycles (1 A g^{-1})	94
3D porous PANI/RGO composite	808 (53.33 A g^{-1}) 3-electrode	5.717 (377.4 mA cm^{-2})	.		95
rGO/PANI composite	438.8 (0.5A g^{-1}) 2-electrode			76.5% capacitance after 2000 cycles (4 A g^{-1})	96
N-3D-rGO/PANI-B	282 (1 A g^{-1}.) 3-electrode			69% capacitance after 1000 cycles (2 A g^{-1})	97

UGA/PANI composites	538 (1 A g^{-1}) 3-electrode			74% capacitance after 1000 cycles (3 A g^{-1})	98
3D-G/PANI composite	567 (1 A g^{-1}) 3-electrode (77.8 Wh Kg^{-1}) 2-electrode		720 3-electrode	100% after 10000 cycles 5 A g^{-1}, 3-electrode 95.6% for 10,000 cycles 2-electrode	99
Graphene/PANI composite nanosheets	665 (1 A g^{-1}). 2-electrode		847	86% capacitance after 1000 cycles (20 A g^{-1})	100
3D RGO/S-PANI aerogel	480 (1 A g^{-1}) 2-electrode			96.14% after 10,000 cycles (10 A g^{-1})	101
RGO/PANI/RGO hybrid paper	581 (1 A g^{-1})			85% capacitance after 10000 cycles (10A g^{-1})	107
GH-PANI/GP	864 (1 A g^{-1}) 2-electrode	190.6 (0.5 mA cm^{-2})		85.6% capacitance after 5000 cycles (8 A g^{-1})	108
PANI/rGO electrodes		1329 2-electrode		75% capacitance after 1000 cycles 50 mA cm^{-2}	109
Graphene/sulfonated PANI (rG/SP)		3.31	16.55 2-electrode	85.4% capacitance after 10000 cycles	110

From Table S1, among 59 papers about composite electrode, 22% of works (13 papers) had a specific capacitance of more than 800 F g^{-1}, and only 6.78% works (four papers) achieved the higher capacitance (≥800 F g^{-1}) in two-electrode systems. From the long-term cycling performance, 16.95% works (10 papers) list the capacitance retention over 10,000 cycles, and only 10.17% works (six papers) have a high capacitance retention (≥85%). Therefore, there is still a long way to further enhance the electrochemical performance of graphene/PANI composite electrodes.

References

1. Chee, W.K.; Lim, H.N.; Zainal, Z.; Huang, N.M.; Harrison, I.; Andou, Y. Flexible Graphene-Based Supercapacitors: A Review. *J. Phys. Chem. C* **2016,** *120*, 4153–4172.
2. Lim, E.; Jo, C.; Lee, J. A mini review of designed mesoporous materials for energy-storage applications: from electric double-layer capacitors to hybrid supercapacitors. *Nanoscale* **2016,** *8*, 7827–7833.
3. Sk, M.M.; Yue, C.Y.; Ghosh, K.; Jena, R.K. Review on advances in porous nanostructured nickel oxides and their composite electrodes for high-performance supercapacitors. *J. Power Sources* **2016**, *308*, 121–140.
4. Eftekhari, A.; Li, L.; Yang, Y, Polyaniline supercapacitors. *J. Power Sources* **2017**, *347*, 86–107.
5. Chauhan, N.P.S.; Mozafari, M.; Chundawat, N.S.; Meghwal, K.; Ameta, R.; Ameta, S.C. High-performance supercapacitors based on polyaniline–graphene nanocomposites: Some approaches, challenges and opportunities. *J. Ind. Eng. Chem.* **2016**, *36*, 13–29.
6. Wang, P.-H.; Wang, T.-L.; Lin, W.-C.; Lin, H.-Y.; Lee, M.-H.; Yang, C.-H. Enhanced Supercapacitor Performance Using Electropolymerization of Self-Doped Polyaniline on Carbon Film. *Nanomaterials* **2018,** *8*, 214.
7. Zhang, Y.-S.; Xu, W.-H.; Yao, W.-T.; Yu, S.-H. Oxidation-Reduction Reaction Driven Approach for Hydrothermal Synthesis of Polyaniline Hollow Spheres with Controllable Size and Shell Thickness. *J. Phys. Chem. C* **2009,** *113*, 8588–8594.
8. Du, P.; Wei, W.; Liu, D.; Kang, H.; Liu, P. Fabrication of hierarchical carbon layer encapsulated polyaniline core-shell structure nanotubes and application in supercapacitors. *Chem. Eng. J.* **2018**, *335*, 373–383.
9. Tabrizi, A.G.; Arsalani, N.; Namazi, H.; Ahadzadeh, I. Vanadium oxide assisted synthesis of polyaniline nanoarrays on graphene oxide sheets and its application in supercapacitors. *Electroanal. Chem.* **2017**, *798*, 34–41.
10. Zhou, K.; He, Y.; Xu, Q.; Zhang, Q.E.; Zhou, A.A.; Lu, Z.; Yang, L.-K.; Jiang, Y.; Ge, D.; Liu, X.Y.; et al. A Hydrogel of Ultrathin Pure Polyaniline Nanofibers: Oxidant-Templating Preparation and Supercapacitor Application. *ACS Nano* **2018,** *12*, 5888–5894.

11. Gawli, Y.; Banerjee, A.; Dhakras, D.; Deo, M.; Bulani, D.; Wadgaonkar, P.; Shelke, M.; Ogale, S. 3D Polyaniline Architecture by Concurrent Inorganic and Organic Acid Doping for Superior and Robust High Rate Supercapacitor Performance. *Sci. Rep.* **2016**, *6*, 21002.
12. Kuo, C.-W.; Kuo, P.-L.; Ho, K.-S.; Hsieh, T.-H.; Chen, S.-J.; Wu, T.-Y.; Huang, Y.-C. Polyaniline Doped with Various Inorganic Acids and Polymeric Acids as Platinum Catalyst Support for Methanol Electro-oxidation. *J. Chin. J. Chin. Chem. Soc.* **2012,** *59*, 1294–1302.
13. Kuo, C.-W.; Yang, C.-C.; Wu, T.-Y. Facile Synthesis of Composite Electrodes Containing Platinum Particles Distributed in Nanowires of Polyaniline-Poly(Acrylic Acid) for Methanol Oxidation. *Int. J. Electrochem. Sci.* **2011**, *6*, 3196–3209.
14. Tabrizi, A.G.; Arsalani, N.; Mohammadi, A.; Ghadimi, L.S.; Ahadzadeh, I.; Namazi, H. A new route for the synthesis of polyaniline nanoarrays on graphene oxide for high-performance supercapacitors. *Electrochim. Acta* **2018**, *265*, 379–390.
15. Yuan, Y.; Zhu, W.; Du, G.; Wang, D.; Zhu, J.; Zhu, X.; Pezzotti, G. Two-step method for synthesizing polyaniline with bimodal nanostructures for high performance supercapacitors. *Electrochim. Acta* **2018**, *282*, 286–294.
16. Yang, M.; Cao, L.; Tan, L. Synthesis of sea urchin-like polystyrene/polyaniline microspheres by seeded swelling polymerization and their catalytic application. *Colloid. Surface A* **2014**, *441*, 678–684.
17. Yang, M.; Yao, X.; Wang, G.; Ding, H. A simple method to synthesize sea urchin-like polyaniline hollow spheres. *Colloid. Surface A* **2008,** *324*, 113–116.
18. Wang, Y.; Xu, S.; Liu, W.; Cheng, H.; Chen, S.; Liu, X.; Liu, J.; Tai, Q.; Hu, C. Facile Fabrication of Urchin-like Polyaniline Microspheres for Electrochemical Energy Storage. *Electrochim. Acta* **2017**, *254*, 25–35.
19. Luo, J.; Ma, Q.; Gu, H.; Zheng, Y.; Liu, X. Three-dimensional graphene-polyaniline hybrid hollow spheres by layer-by-layer assembly for application in supercapacitor. *Electrochim. Acta* **2015**, *173*, 184–192.
20. Trung, N.B.; Tam, T.V.; Kim, H.R.; Hur, S.H.; Kim, E.J.; Choi, W.M. Three-dimensional hollow balls of graphene-polyaniline hybrids for supercapacitor applications. *Chem. Eng. J.* **2014**, *255*, 89–96.
21. Liu, X.; Wen, N.; Wang, X.; Zheng, Y. A High-Performance Hierarchical Graphene@Polyaniline@Graphene Sandwich Containing Hollow Structures for Supercapacitor Electrodes. *ACS. Sustain. Chem. Eng.* **2015,** *3*, 475–482.
22. 22. Wang, Q.; Yan, J.; Fan, Z.; Wei, T.; Zhang, M.; Jing, X. Mesoporous polyaniline film on ultra-thin graphene sheets for high performance supercapacitors. *J. Power Sources* **2014**, *247*, 197–203.
23. Tabar, F.A.; Sharif, F.; Mazinani, S. Preparation and electrochemical performance of a novel three dimensional structure of polyaniline hollow fibers decorated by graphene. *Polymer* **2018**, *154*, 80–89.
24. Ma, Y.; Hou, C.; Zhang, H.; Qiao, M.; Chen, Y.; Zhang, H.; Zhang, Q.; Guo, Z. Morphology-dependent electrochemical supercapacitors in multi-dimensional polyaniline nanostructures. *J. Mater. Chem. A* **2017,** *5*, 14041–14052.
25. Guo, L.; Ma, M.; Zhang, N.; Langer, R.; Anderson, D.G. Stretchable Polymeric Multielectrode Array for Conformal Neural Interfacing. *Adv. Mater.* **2014,** *26*, 1427–1433.
26. Li, W.; Gao, F.; Wang, X.; Zhang, N.; Ma, M. Strong and Robust Polyaniline-Based Supramolecular Hydrogels for Flexible Supercapacitors. *Angew. Chem.* **2016,** *128*, 9342–9347.
27. Huang, J.; Li, Q.; Wang, Y.; Wang, Y.; Dong, L.; Xie, H.; Xiong, C. Self-Suspended Polyaniline Doped with a Protonic Acid Containing a Polyethylene Glycol Segment. *Chem. Asian. J.* **2011,** *6*, 2920–2924.
28. Li, H.; Song, J.; Wang, L.; Feng, X.; Liu, R.; Zeng, W.; Huang, Z.; Ma, Y.; Wang, L. Flexible all-solid-state supercapacitors based on polyaniline orderly nanotubes array. *Nanoscale* **2017,** *9*, 193–200.
29. Xing, J.; Liao, M.; Zhang, C.; Yin, M.; Li, D.; Song, Y. The effect of anions on the electrochemical properties of polyaniline for supercapacitors. *Phys. Chem. Chem. Phys.* **2017,** *19*, 14030–14041.
30. Zhang, X.; Meng, X.; Wang, Q.; Qin, B.; Jin, L. e.; Cao, Q. Preparation and electrochemical investigation of polyaniline nanowires for high performance supercapacitor. *Mater. Lett.* **2018**, *217*, 312–315.
31. Shabani-Nooshabadi, M.; Zahedi, F. Electrochemical reduced graphene oxide-polyaniline as effective nanocomposite film for high-performance supercapacitor applications. *Electrochim. Acta* **2017**, *245*, 575–586.
32. Yu, J.; Xie, F.; Wu, Z.; Huang, T.; Wu, J.; Yan, D.; Huang, C.; Li, L. Flexible metallic fabric supercapacitor based on graphene/polyaniline composites. *Electrochim. Acta* **2018**, *259*, 968–974.
33. Huang, J.; Kaner, R.B. A General Chemical Route to Polyaniline Nanofibers. *J. Am. Chem. Soc.* **2004,** *126*, 851–855.
34. Souza, V.H.R.; Oliveira, M.M.; Zarbin, A.J.G. Bottom-up synthesis of graphene/polyaniline nanocomposites for flexible and transparent energy storage devices. *J. Power Sources* **2017**, *348*, 87–93.
35. Feng, X.; Chen, N.; Zhou, J.; Li, Y.; Huang, Z.; Zhang, L.; Ma, Y.; Wang, L.; Yan, X. Facile synthesis of shape-controlled graphene-polyaniline composites for high performance supercapacitor electrode materials. *New. J. Chem.* **2015,** *39*, 2261–2268.
36. Guo, F.; Liu, Q.; Mi, H. Flexible and cross-linked polyaniline nets as promising supercapacitor electrodes. *Mater. Lett.* **2016**, *163*, 115–117.
37. Xu, H.; Li, X.; Wang, G. Polyaniline nanofibers with a high specific surface area and an improved pore structure for supercapacitors. *J. Power Sources* **2015**, *294*, 16–21.
38. Zhang, K.; Zhang, L.L.; Zhao, X.S.; Wu, J. Graphene/Polyaniline Nanofiber Composites as Supercapacitor Electrodes. *Chem. Mater.* **2010,** *22*, 1392–1401.

39. Salunkhe, R.R.; Hsu, S.-H.; Wu, K.C.W.; Yamauchi, Y. Large-Scale Synthesis of Reduced Graphene Oxides with Uniformly Coated Polyaniline for Supercapacitor Applications. *ChemSusChem* **2014**, *7*, 1551–1556.
40. Zhang, Y.; Si, L.; Zhou, B.; Zhao, B.; Zhu, Y.; Zhu, L.; Jiang, X. Synthesis of novel graphene oxide/pristine graphene/polyaniline ternary composites and application to supercapacitor. *Chem. Eng. J.* **2016**, *288*, 689–700.
41. Jiang, F.; Chen, D.; Li, R.; Wang, Y.; Zhang, G.; Li, S.; Zheng, J.; Huang, N.; Gu, Y.; Wang, C.; et al. Eco-friendly synthesis of size-controllable amine-functionalized graphene quantum dots with antimycoplasma properties. *Nanoscale* **2013**, *5*, 1137–1142.
42. Mondal, S.; Rana, U.; Malik, S. Graphene quantum dot-doped polyaniline nanofiber as high performance supercapacitor electrode materials. *Chem. Commun.* **2015**, *51*, 12365–12368.
43. Cao, H.; Zhou, X.; Zhang, Y.; Chen, L.; Liu, Z. Microspherical polyaniline/graphene nanocomposites for high performance supercapacitors. *J. Power Sources* **2013**, *243*, 715–720.
44. Yu, T.; Zhu, P.; Xiong, Y.; Chen, H.; Kang, S.; Luo, H.; Guan, S. Synthesis of microspherical polyaniline/ graphene composites and their application in supercapacitors. *Electrochim. Acta* **2016**, *222*, 12–19.
45. Chang, T.-W.; Lin, L.-Y.; Peng, P.-W.; Zhang, Y.X.; Huang, Y.-Y. Enhanced electrocapacitive performance for the supercapacitor with tube-like polyaniline and graphene oxide composites. *Electrochim. Acta* **2018**, *259*, 348–354.
46. Liu, X.; Zheng, Y.; Wang, X. Controllable Preparation of Polyaniline-Graphene Nanocomposites using Functionalized Graphene for Supercapacitor Electrodes. *Chem. Eur. J.* **2015**, *21*, 10408–10415.
47. Zou, Y.; Zhang, Z.; Zhong, W.; Yang, W. Hydrothermal direct synthesis of polyaniline, graphene/polyaniline and N-doped graphene/polyaniline hydrogels for high performance flexible supercapacitors. *J. Mater. Chem. A* **2018**, *6*, 9245–9256.
48. Zou, Y.; Liu, R.; Zhong, W.; Yang, W. Mechanically robust double-crosslinked network functionalized graphene/polyaniline stiff hydrogels for superior performance supercapacitors. *J. Mater. Chem. A* **2018**, *6*, 8568–8578.
49. Kumar, N.A.; Choi, H.-J.; Shin, Y.R.; Chang, D.W.; Dai, L.; Baek, J.-B. Polyaniline-Grafted Reduced Graphene Oxide for Efficient Electrochemical Supercapacitors. *ACS Nano* **2012**, *6*, 1715–1723.
50. Bai, H.; Xu, Y.; Zhao, L.; Li, C.; Shi, G. Non-covalent functionalization of graphene sheets by sulfonated polyaniline. *Chem. Commun.* **2009**, *13*, 1667–1669.
51. Liu, Y.; Ma, Y.; Guang, S.; Ke, F.; Xu, H. Polyaniline-graphene composites with a three-dimensional array-based nanostructure for high-performance supercapacitors. *Carbon* **2015**, *83*, 79–89.
52. Li, Z.-F.; Zhang, H.; Liu, Q.; Liu, Y.; Stanciu, L.; Xie, J. Covalently-grafted polyaniline on graphene oxide sheets for high performance electrochemical supercapacitors. *Carbon* **2014**, *71*, 257–267.
53. Gao, Z.; Wang, F.; Chang, J.; Wu, D.; Wang, X.; Wang, X.; Xu, F.; Gao, S.; Jiang, K. Chemically grafted graphene-polyaniline composite for application in supercapacitor. *Electrochim. Acta* **2014**, *133*, 325–334.
54. Luo, J.; Zhong, W.; Zou, Y.; Xiong, C.; Yang, W. Preparation of morphology-controllable polyaniline and polyaniline/graphene hydrogels for high performance binder-free supercapacitor electrodes. *J. Power Sources* **2016**, *319*, 73–81.
55. Bulin, C.; Yu, H.; Ge, X.; Xin, G.; Xing, R.; Li, R.; Zhang, B. Preparation and supercapacitor performance of functionalized graphene aerogel loaded with polyaniline as a freestanding electrode. *J. Mater. Sci.* **2017**, *52*, 5871–5881.
56. Lu, X.; Li, L.; Song, B.; Moon, K.-S.; Hu, N.; Liao, G.; Shi, T.; Wong, C. Mechanistic investigation of the graphene functionalization using p-phenylenediamine and its application for supercapacitors. *Nano Energy* **2015**, *17*, 160–170.
57. Li, L.; Song, B.; Maurer, L.; Lin, Z.; Lian, G.; Tuan, C.-C.; Moon, K.-S.; Wong, C.-P. Molecular engineering of aromatic amine spacers for high-performance graphene-based supercapacitors. *Nano Energy* **2016**, *21*, 276–294.
58. Zou, Y.; Zhong, W.; Li, S.; Luo, J.; Xiong, C.; Yang, W. Structure of functionalized nitrogen-doped graphene hydrogels derived from isomers of phenylenediamine and graphene oxide based on their high electrochemical performance. *Electrochim. Acta* **2016**, *212*, 828–838.
59. Ke, F.; Liu, Y.; Xu, H.; Ma, Y.; Guang, S.; Zhang, F.; Lin, N.; Ye, M.; Lin, Y.; Liu, X. Flower-like polyaniline/graphene hybrids for high-performance supercapacitor. *Compos. Sci. Technol.* **2017**, *142*, 286–293.
60. Jin, K.; Zhang, W.; Wang, Y.; Guo, X.; Chen, Z.; Li, L.; Zhang, Y.; Wang, Z.; Chen, J.; Sun, L.; et al. In-situ hybridization of polyaniline nanofibers on functionalized reduced graphene oxide films for high-performance supercapacitor. *Electrochim. Acta* **2018**, *285*, 221–229.
61. Liu, Y.; Ma, Y.; Guang, S.; Xu, H.; Su, X. Facile fabrication of three-dimensional highly ordered structural polyaniline-graphene bulk hybrid materials for high performance supercapacitor electrodes. *J. Mater. Chem. A* **2014**, *2*, 813–823.
62. Hu, R.; Zhao, J.; Zhu, G.; Zheng, J. Fabrication of flexible free-standing reduced graphene oxide/polyaniline nanocomposite film for all-solid-state flexible supercapacitor. *Electrochim. Acta* **2018**, *261*, 151–159.
63. Kumari, P.; Khawas, K.; Nandy, S.; Kuila, B.K. A supramolecular approach to Polyaniline graphene nanohybrid with three dimensional pillar structures for high performing electrochemical supercapacitor applications. *Electrochim. Acta* **2016**, *190*, 596–604.
64. Wu, Q.; Xu, Y.; Yao, Z.; Liu, A.; Shi, G. Supercapacitors Based on Flexible Graphene/Polyaniline Nanofiber Composite Films. *ACS Nano* **2010**, *4*, 1963–1970.
65. Du, P.; Liu, H.C.; Yi, C.; Wang, K.; Gong, X. Polyaniline-Modified Oriented Graphene Hydrogel Film as the Free-Standing Electrode for Flexible Solid-State Supercapacitors. *Acs. Appl. Mater. Inter.* **2015**, *7*, 23932–23940.

66. Zheng, W.; Lv, R.; Na, B.; Liu, H.; Jin, T.; Yuan, D. Nanocellulose-mediated hybrid polyaniline electrodes for high performance flexible supercapacitors. *J. Mater. Chem. A* **2017**, *5*, 12969–12976.
67. Iqbal, N.; Wang, X.; Babar, A.A.; Yan, J.; Yu, J.; Park, S.-J.; Ding, B. Polyaniline Enriched Flexible Carbon Nanofibers with Core–Shell Structure for High-Performance Wearable Supercapacitors. *Adv. Mater. Interfaces* **2017**, *4*, 1700855.
68. Cong, H.-P.; Ren, X.-C.; Wang, P.; Yu, S.-H. Flexible graphene–polyaniline composite paper for high-performance supercapacitor. *Energ. Environ. Sci.* **2013**, *6*, 1185–1191.
69. Zang, X.; Li, X.; Zhu, M.; Li, X.; Zhen, Z.; He, Y.; Wang, K.; Wei, J.; Kang, F.; Zhu, H. Graphene/polyaniline woven fabric composite films as flexible supercapacitor electrodes. *Nanoscale* **2015**, *7*, 7318–7322.
70. Wang, S.; Ma, L.; Gan, M.; Fu, S.; Dai, W.; Zhou, T.; Sun, X.; Wang, H.; Wang, H. Free-standing 3D graphene/polyaniline composite film electrodes for high-performance supercapacitors. *J. Power Sources* **2015**, *299*, 347–355.
71. Khosrozadeh, A.; Darabi, M.A.; Xing, M.; Wang, Q. Flexible Electrode Design: Fabrication of Freestanding Polyaniline-Based Composite Films for High-Performance Supercapacitors. *Acs. Appl. Mater. Inter.* **2016**, *8*, 11379–11389.
72. Xin, G.; Wang, Y.; Liu, X.; Zhang, J.; Wang, Y.; Huang, J.; Zang, J. Preparation of self-supporting graphene on flexible graphite sheet and electrodeposition of polyaniline for supercapacitor. *Electrochim. Acta* **2015**, *167*, 254–261.
73. Lin, Y.; Zhang, H.; Deng, W.; Zhang, D.; Li, N.; Wu, Q.; He, C. In-situ growth of high-performance all-solid-state electrode for flexible supercapacitors based on carbon woven fabric/polyaniline/graphene composite. *J. Power Sources* **2018**, *384*, 278–286.
74. Ji, H.; Zhang, L.; Pettes, M.T.; Li, H.; Chen, S.; Shi, L.; Piner, R.; Ruoff, R.S. Ultrathin Graphite Foam: A Three-Dimensional Conductive Network for Battery Electrodes. *Nano. Lett.* **2012**, *12*, 2446–2451.
75. Yu, M.; Ma, Y.; Liu, J.; Li, S. Polyaniline nanocone arrays synthesized on three-dimensional graphene network by electrodeposition for supercapacitor electrodes. *Carbon* **2015**, *87*, 98–105.
76. Kulkarni, S.B.; Patil, U.M.; Shackery, I.; Sohn, J.S.; Lee, S.; Park, B.; Jun, S. High-performance supercapacitor electrode based on a polyaniline nanofibers/3D graphene framework as an efficient charge transporter. *J. Mater. Chem. A* **2014**, *2*, 4989–4998.
77. Pedrós, J.; Boscá, A.; Martínez, J.; Ruiz-Gómez, S.; Pérez, L.; Barranco, V.; Calle, F. Polyaniline nanofiber sponge filled graphene foam as high gravimetric and volumetric capacitance electrode. *J. Power Sources* **2016**, *317*, 35–42.
78. Xie, Y.; Liu, Y.; Zhao, Y.; Tsang, Y.H.; Lau, S.P.; Huang, H.; Chai, Y. Stretchable all-solid-state supercapacitor with wavy shaped polyaniline/graphene electrode. *J. Mater. Chem. A* **2014**, *2*, 9142–9149.
79. Yu, P.; Zhao, X.; Huang, Z.; Li, Y.; Zhang, Q. Free-standing three-dimensional graphene and polyaniline nanowire arrays hybrid foams for high-performance flexible and lightweight supercapacitors. *J. Mater. Chem. A* **2014**, *2*, 14413–14420.
80. Hong, X.; Lu, Y.; Li, S.; Wang, X.; Wang, X.; Liang, J. Carbon foam@reduced graphene oxide scaffold grown with polyaniline nanofibers for high performance symmetric supercapacitor. *Electrochim. Acta* **2019**, *294*, 376–382.
81. Wu, L.; Hao, L.; Pang, B.; Wang, G.; Zhang, Y.; Li, X. MnO_2 nanoflowers and polyaniline nanoribbons grown on hybrid graphene/Ni 3D scaffolds by in situ electrochemical techniques for high-performance asymmetric supercapacitors. *J. Mater. Chem. A* **2017**, *5*, 4629–4637.
82. Yu, P.; Zhao, X.; Li, Y.; Zhang, Q. Controllable growth of polyaniline nanowire arrays on hierarchical macro/mesoporous graphene foams for high-performance flexible supercapacitors. *Appl. Surf. Sci.* **2017**, *393*, 37–45.
83. Zhang, L.; Shi, G. Preparation of Highly Conductive Graphene Hydrogels for Fabricating Supercapacitors with High Rate Capability. *J. Phys. Chem. C* **2011**, *115*, 17206–17212.
84. Gao, S.; Zhang, L.; Qiao, Y.; Dong, P.; Shi, J.; Cao, S. Electrodeposition of polyaniline on three-dimensional graphene hydrogel as a binder-free supercapacitor electrode with high power and energy densities. *RSC. Adv.* **2016**, *6*, 58854–58861.
85. Zhao, T.; Ji, X.; Bi, P.; Jin, W.; Xiong, C.; Dang, A.; Li, H.; Li, T.; Shang, S.; Zhou, Z. In situ synthesis of interlinked three-dimensional graphene foam/polyaniline nanorod supercapacitor. *Electrochim. Acta* **2017**, *230*, 342–349.
86. Zhang, L.; Huang, D.; Hu, N.; Yang, C.; Li, M.; Wei, H.; Yang, Z.; Su, Y.; Zhang, Y. Three-dimensional structures of graphene/polyaniline hybrid films constructed by steamed water for high-performance supercapacitors. *J. Power Sources* **2017**, *342*, 1–8.
87. Wang, J.; Xian, H.; Peng, T.; Sun, H.; Zheng, F. Three-dimensional graphene-wrapped PANI nanofiber composite as electrode material for supercapacitors. *RSC Adv.* **2015**, *5*, 13607–13612.
88. Qu, Y.; Lu, C.; Su, Y.; Cui, D.; He, Y.; Zhang, C.; Cai, M.; Zhang, F.; Feng, X.; Zhuang, X. Hierarchical- graphene-coupled polyaniline aerogels for electrochemical energy storage. *Carbon* **2018**, *127*, 77–84.
89. Van Hoa, N.; Quyen, T.T.H.; Van Hieu, N.; Ngoc, T.Q.; Thinh, P.V.; Dat, P.A.; Nguyen, H.T.T. Three-dimensional reduced graphene oxide-grafted polyaniline aerogel as an active material for high performance supercapacitors. *Synthetic. Met.* **2017**, *223*, 192–198.
90. Wang, R.; Han, M.; Zhao, Q.; Ren, Z.; Guo, X.; Xu, C.; Hu, N.; Lu, L. Hydrothermal synthesis of nanostructured graphene/polyaniline composites as high-capacitance electrode materials for supercapacitors. *Sci. Rep.* **2017**, *7*, 44562.
91. Ates, M.; El-Kady, M.; Kaner, R.B. Three-dimensional design and fabrication of reduced graphene oxide/polyaniline composite hydrogel electrodes for high performance electrochemical supercapacitors. *Nanotechnology* **2018**, *29*, 175402.
92. Zheng, X.; Yu, H.; Xing, R.; Ge, X.; Sun, H.; Li, R.; Zhang, Q. Multi-growth site graphene/polyaniline composites with highly enhanced specific capacitance and rate capability for supercapacitor application. *Electrochim. Acta* **2018**, *260*, 504–513.

93. Zhu, J.; Kong, L.; Shen, X.; Chen, Q.; Ji, Z.; Wang, J.; Xu, K.; Zhu, G. Three-dimensional N-doped graphene/ polyaniline composite foam for high performance supercapacitors. *Appl. Surf. Sci.* **2018**, *428*, 348–355.
94. Tang, W.; Peng, L.; Yuan, C.; Wang, J.; Mo, S.; Zhao, C.; Yu, Y.; Min, Y.; Epstein, A.J. Facile synthesis of 3D reduced graphene oxide and its polyaniline composite for super capacitor application. *Synthetic. Met.* **2015**, *202*, 140–146.
95. Wu, J.; Zhang, Q. e.; Wang, J.; Huang, X.; Bai, H. A self-assembly route to porous polyaniline/reduced graphene oxide composite materials with molecular-level uniformity for high-performance supercapacitors. *Energy Environ. Sci.* **2018,** *11*, 1280–1286.
96. Hong, X.; Zhang, B.; Murphy, E.; Zou, J.; Kim, F. Three-dimensional reduced graphene oxide/polyaniline nanocomposite film prepared by diffusion driven layer-by-layer assembly for high-performance supercapacitors. *J. Power Sources* **2017**, *343*, 60–66.
97. Liu, Z.; Li, D.; Li, Z.; Liu, Z.; Zhang, Z. Nitrogen-doped 3D reduced graphene oxide/polyaniline composite as active material for supercapacitor electrodes. *Appl. Surf. Sci.* **2017**, *422*, 339–347.
98. Wu, X.; Tang, L.; Zheng, S.; Huang, Y.; Yang, J.; Liu, Z.; Yang, W.; Yang, M. Hierarchical unidirectional graphene aerogel/polyaniline composite for high performance supercapacitors. *J. Power Sources* **2018**, *397*, 189–195.
99. Li, K.; Huang, Y.; Liu, J.; Sarfraz, M.; Agboola, P.O.; Shakir, I.; Xu, Y. A three-dimensional graphene framework-enabled high-performance stretchable asymmetric supercapacitor. *J. Mater. Chem. A* **2018,** *6*, 1802–1808.
100. Li, K.; Liu, J.; Huang, Y.; Bu, F.; Xu, Y. Integration of ultrathin graphene/polyaniline composite nanosheets with a robust 3D graphene framework for highly flexible all-solid-state supercapacitors with superior energy density and exceptional cycling stability. *J. Mater. Chem. A* **2017,** *5*, 5466–5474.
101. Gao, Z.; Yang, J.; Huang, J.; Xiong, C.; Yang, Q. A three-dimensional graphene aerogel containing solvent-free polyaniline fluid for high performance supercapacitors. *Nanoscale* **2017,** *9*, 17710–17716.
102. Gupta, S.; Price, C. Investigating graphene/conducting polymer hybrid layered composites as pseudocapacitors: Interplay of heterogeneous electron transfer, electric double layers and mechanical stability. *Compos. Part B-Eng*. **2016**, *105*, 46–59.
103. Zou, J.; Kim, F. Diffusion driven layer-by-layer assembly of graphene oxide nanosheets into porous three-dimensional macrostructures. *Nat. Commun.* **2014**, *5*, 5254.
104. Zou, J.; Zhang, B.; Murphy, E.; Kim, F. Application of Diffusion-Driven Layer-by-Layer Assembly for Fabricating Compact Graphene-Based Supercapacitors. *Adv. Mater. Interfaces* **2016,** *3*, 1600260.
105. MacDonald, E.; Wicker, R. Multiprocess 3D printing for increasing component functionality. *Science* **2016,** *353*, 2093.
106. Ambrosi, A.; Pumera, M. 3D-printing technologies for electrochemical applications. *Chem. Soc. Rev.* **2016,** *45*, 2740–2755.
107. Xiao, F.; Yang, S.; Zhang, Z.; Liu, H.; Xiao, J.; Wan, L.; Luo, J.; Wang, S.; Liu, Y. Scalable Synthesis of Freestanding Sandwich-structured Graphene/Polyaniline/Graphene Nanocomposite Paper for Flexible All-Solid-State Supercapacitor. *Sci. Rep.* **2015**, *5*, 9359.
108. Chi, K.; Zhang, Z.; Xi, J.; Huang, Y.; Xiao, F.; Wang, S.; Liu, Y. Freestanding Graphene Paper Supported Three-Dimensional Porous Graphene-Polyaniline Nanocomposite Synthesized by Inkjet Printing and in Flexible All-Solid-State Supercapacitor. *ACS. Appl. Mater. Inter.* **2014,** *6*, 16312–16319.
109. Wang, Z.; Zhang, Q.E.; Long, S.; Luo, Y.; Yu, P.; Tan, Z.; Bai, J.; Qu, B.; Yang, Y.; Shi, J.; et al. Three-Dimensional Printing of Polyaniline/Reduced Graphene Oxide Composite for High-Performance Planar Supercapacitor. *ACS. Appl. Mater. Inter.* **2018,** *10*, 10437–10444.
110. Song, B.; Li, L.; Lin, Z.; Wu, Z.-K.; Moon, K.-S.; Wong, C.-P. Water-dispersible graphene/polyaniline composites for flexible micro-supercapacitors with high energy densities. *Nano Energy* **2015**, *16*, 470–478.
